# Recombinant fibrous protein biomaterials meet skin tissue engineering

**DOI:** 10.3389/fbioe.2024.1411550

**Published:** 2024-08-14

**Authors:** Dipeng Li, Yirong Wang, Shan Zhu, Xuezhong Hu, Renjie Liang

**Affiliations:** ^1^ Hangzhou Ninth People’s Hospital, Hangzhou, China; ^2^ Hangzhou Singclean Medical Products Co., Ltd., Hangzhou, China; ^3^ Affiliated Cixi Hospital, Wenzhou Medical University, Ningbo, China; ^4^ School of Medicine, Southeast University, Nanjing, China

**Keywords:** recombinant protein, collagen, elastin, silk, skin tissue engineering

## Abstract

Natural biomaterials, particularly fibrous proteins, are extensively utilized in skin tissue engineering. However, their application is impeded by batch-to-batch variance, limited chemical or physical versatility, and environmental concerns. Recent advancements in gene editing and fermentation technology have catalyzed the emergence of recombinant fibrous protein biomaterials, which are gaining traction in skin tissue engineering. The modular and highly customizable nature of recombinant synthesis enables precise control over biomaterial design, facilitating the incorporation of multiple functional motifs. Additionally, recombinant synthesis allows for a transition from animal-derived sources to microbial sources, thereby reducing endotoxin content and rendering recombinant fibrous protein biomaterials more amenable to scalable production and clinical use. In this review, we provide an overview of prevalent recombinant fibrous protein biomaterials (collagens, elastin, silk proteins and their chimeric derivatives) used in skin tissue engineering (STE) and compare them with their animal-derived counterparts. Furthermore, we discuss their applications in STE, along with the associated challenges and future prospects.

## Graphical Abstract

**Figure:**
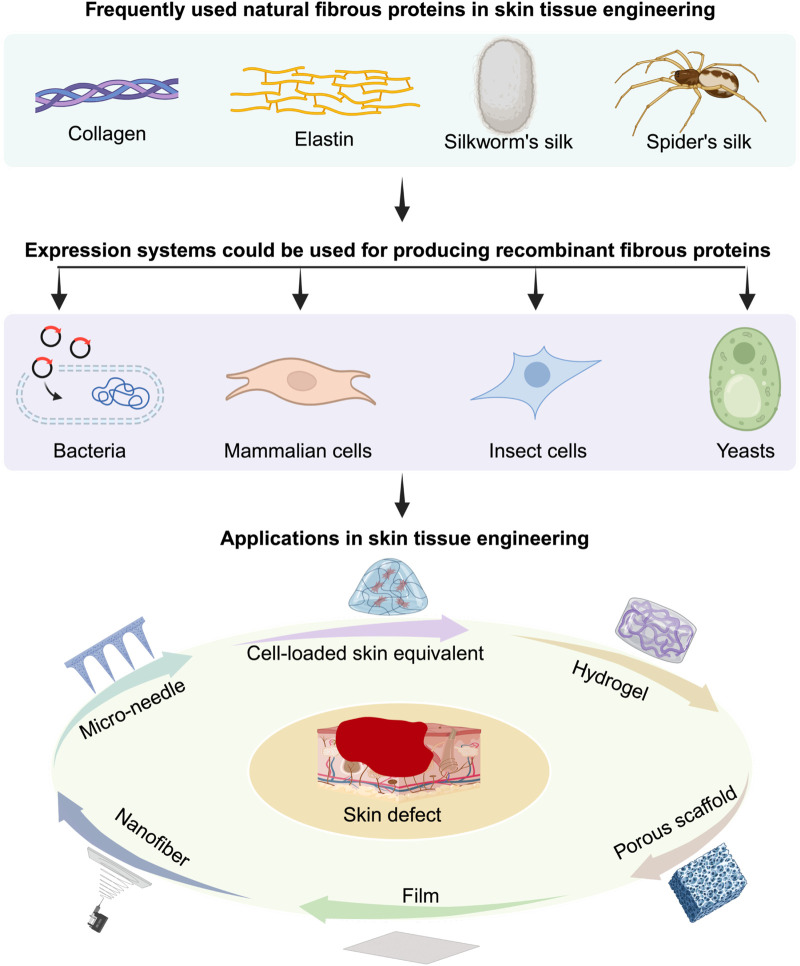
In this review, the frequently used recombinant fibrous proteins are summarized and their applications in skin tissue engineering are discussed. Besides, the future challenges and opportunities in this area are also discussed.

## 1 Introduction

Skin plays a crucial role in protecting the human body against environmental factors, dehydration, and infections. However, significant skin loss resulting from burns, wounds, or tumor resection can severely impact people’s quality of life. While skin transplantation has been a longstanding treatment for skin defects, the shortage of transplantable skin and the risk of immune rejection have spurred the development of new technologies (Zhou et al., 2013; Arabi et al., 2023). Tissue engineering has emerged as a solution to address these challenges. Broadly speaking, tissue engineering involves the use of biomaterial scaffolds, with or without cells and growth factors, to facilitate tissue regeneration (Arif et al., 2022; Li et al., 2022; Norahan et al., 2023). These scaffolds should provide mechanical support, cell adhesion sites, and ideally degrade at a rate conducive to skin regeneration (Qin et al., 2022; Balavigneswaran et al., 2023). Therefore, natural biomaterials with excellent biocompatibility and biodegradability have found extensive applications in skin tissue engineering (STE).

For decades, natural fibrous protein biomaterials, such as collagens, elastin, and silk proteins, have been widely utilized in STE, primarily sourced from animal tissues or products (Li Z.-H. et al., 2013; Bakhshandeh et al., 2021; Akdag et al., 2023; De Giorgio et al., 2024; Gardeazabal and Izeta, 2024; Karahisar Turan et al., 2024; La Monica et al., 2024; Santos et al., 2024). However, concerns regarding the complex extraction process, batch-to-batch variation, cost, and biosafety have prompted the exploration of alternative approaches (Bakhshandeh et al., 2021; Cao et al., 2024a). With advances in genetics, particularly in cross-species gene editing and expression methods, scientists have pursued new methods for producing these natural fibrous protein biomaterials to overcome these challenges (Foo and Kaplan, 2002; Annabi et al., 2013; Yigit et al., 2016; Incir and Kaplan, 2024; Wang et al., 2024). The increasing demand for scalable production, easy quality control, and cost-effectiveness has ushered in a new era—the era of recombinant biomaterials (Cao et al., 2024a; Bitar et al., 2024; Guo et al., 2024). Notably, fibrin and keratin are another two fibrous proteins that have been used in biomedical research regarding skin. However, recombinant fibrin and keratin have not been widely studied in STE. Thus, this review will not discuss about them; more details could be found in the relevant references (Kljenak et al., 2016; Park and Woo, 2018; Parker et al., 2020; Ledford et al., 2022).

Recombinant biomaterials, produced using synthetic biology techniques, involve the rational genetic engineering of microorganisms, such as *Escherichia coli* (*Escherichia coli*), yeast, and mammalian cells, to produce desired materials (Wang et al., 2024). Actually, animals, plants and insects can also be genetically modified to become expressing systems (John et al., 1999; Toman et al., 1999; Moire et al., 2003; Xu, 2014; Sharma et al., 2017; Mortimer, 2019; Burnett and Burnett, 2020; Shanmugaraj et al., 2020; Schillberg and Finnern, 2021); here, we only focus on non-animal and non-plant expression systems due to their fast growing, easy delivering and safety features. Utilizing recombinant DNA technology, scientists can create new materials by combining genetic information from different sources, often incorporating biological components (Hayashi et al., 2001; Shaoping et al., 2005; Carvalho et al., 2008; Cabanne et al., 2009; Plowright et al., 2016). Compared to naturally extracted biomaterials, recombinant biomaterials offer advantages in batch-to-batch stability and physical and chemical versatility (Li L. et al., 2013; Qian et al., 2020; Chang et al., 2021). Additionally, recombinant biomaterials possess strengths over synthetic biomaterials, including biodegradability and bio-activity.

Many recombinant fibrous protein biomaterials, such as recombinant collagens, elastin, and silk proteins, have already been widely employed in STE (Yang et al., 2004; Wise et al., 2009; Annabi et al., 2013; Low et al., 2015; Zhang et al., 2015; Chouhan et al., 2018; Davison-Kotler et al., 2019; Chouhan and Mandal, 2020; Salehi et al., 2020; Chen et al., 2021; Sarangthem et al., 2021). Besides, the highly customizable and modular design of these biomaterials makes them promising candidates for STE (DiMarco and Heilshorn, 2012). These proteins can be combined via gene constructs encoding at least two types of natural proteins, such as elastin and silk fibroin, resulting in the recombinant chimeric proteins (Machado et al., 2013). Thus, unlike extracted natural protein biomaterials, recombinant protein biomaterials empower researchers to fine-tune properties such as biofunctions, biocompatibility, mechanical strength, and degradation rates. The precision offered by genetic engineering enables the creation of biomaterials that closely mimic natural skin tissues or possess entirely novel characteristics. As we explore the landscape of recombinant fibrous protein biomaterials used in STE, this review aims to provide a comprehensive overview of the current state of the field, highlighting key developments, challenges, and future prospects.

## 2 Current recombinant fibrous proteins used in STE

Recombinant fibrous protein biomaterials could be obtained with different kinds of expression systems, such as mammalian cells, *Escherichia coli* (*E. coli*), yeasts and cultured insect cells (Kaur et al., 2018a; Tripathi and Shrivastava, 2019; Karbalaei et al., 2020a). For this review, we only focus on recombinant fibrous proteins from these single-cell unit expression systems. These expression systems can be categorized into prokaryotic and eukaryotic expression systems. The commonly used prokaryotic expression system (e.g., *Escherichia coli*) has the advantages of fast proliferation, simple nutritional requirements, high expression level, and easy for scalable production, etc (Kaur et al., 2018b; Karbalaei et al., 2020b). However, prokaryotic expression system has its own limitations, such as intracellular misfolding of heterologous proteins, production of lipopolysaccharide (Rosano and Ceccarelli, 2014a). In addition, the lack of post-translational modification mechanisms is another disadvantage of prokaryotic expression systems (Sahdev et al., 2008). On the contrary, eukaryotic expression systems, such as mammalian cell and insect cell systems that allow for proper protein folding, post-translational modifications, and glycosylation of recombinant proteins at the correct sites (Yang et al., 2023). Nevertheless, since protein secretion in eukaryotic cells requires a series of complex processes such as signal peptide (SP) cleavage, endoplasmic reticulum (ER) folding, Golgi processing, and vesicular transport, it leads to longer period and inefficient protein secretion, thus affecting the yield of protein (Zhou et al., 2018; Xu et al., 2022). The yeast expression system, as an emerging exogenous protein expression system, combines the advantages of both prokaryotic and eukaryotic expression systems. It can express proteins at a high level and has post-translational modifications (Karbalaei et al., 2020b). However, the glycosylation remains distinct from mammalian cells and yeasts are only suitable for homotrimers (Vaughan et al., 1998; He et al., 2015; Cankorur-Cetinkaya et al., 2018). In the following part, each recombinant fibrous protein will be introduced with their frequently used expression systems and the used genetic information or the sequence of ultimate products.

Table 1 summarizes the advantages and disadvantages of frequently used expression systems for the production of recombinant fibrous proteins.

**TABLE 1:** A comparison of the expression systems for production of recombinant fibrous proteins.

Expression system	Cost	Advantage	Disadvantage	Refs
*Escherichia coli*	Low	Fast proliferation; simple nutritional requirements; high yield	Protein misfolding; lack of post-translational modification; easily forming inclusion bodies; low molecular weight	Kaur et al. (2018b), Karbalaei et al. (2020b)
Mammalian cells	High	Protein folding; post-translational modification; glycosylation	Long period; low yield; virus infection	Geisler and Jarvis (2018), Xu et al. (2022), Yang et al. (2023)
Insect cells	High			
Yeasts	Low	Fast proliferation; high yield; simple culture conditions; extensive post-translational modifications	Glycosylation is different from mammalian cells; only suitable for homotrimers	Vaughan et al. (1998), He et al. (2015), Karbalaei et al. (2020b)

### 2.1 Collagen

**FIGURE 1:**
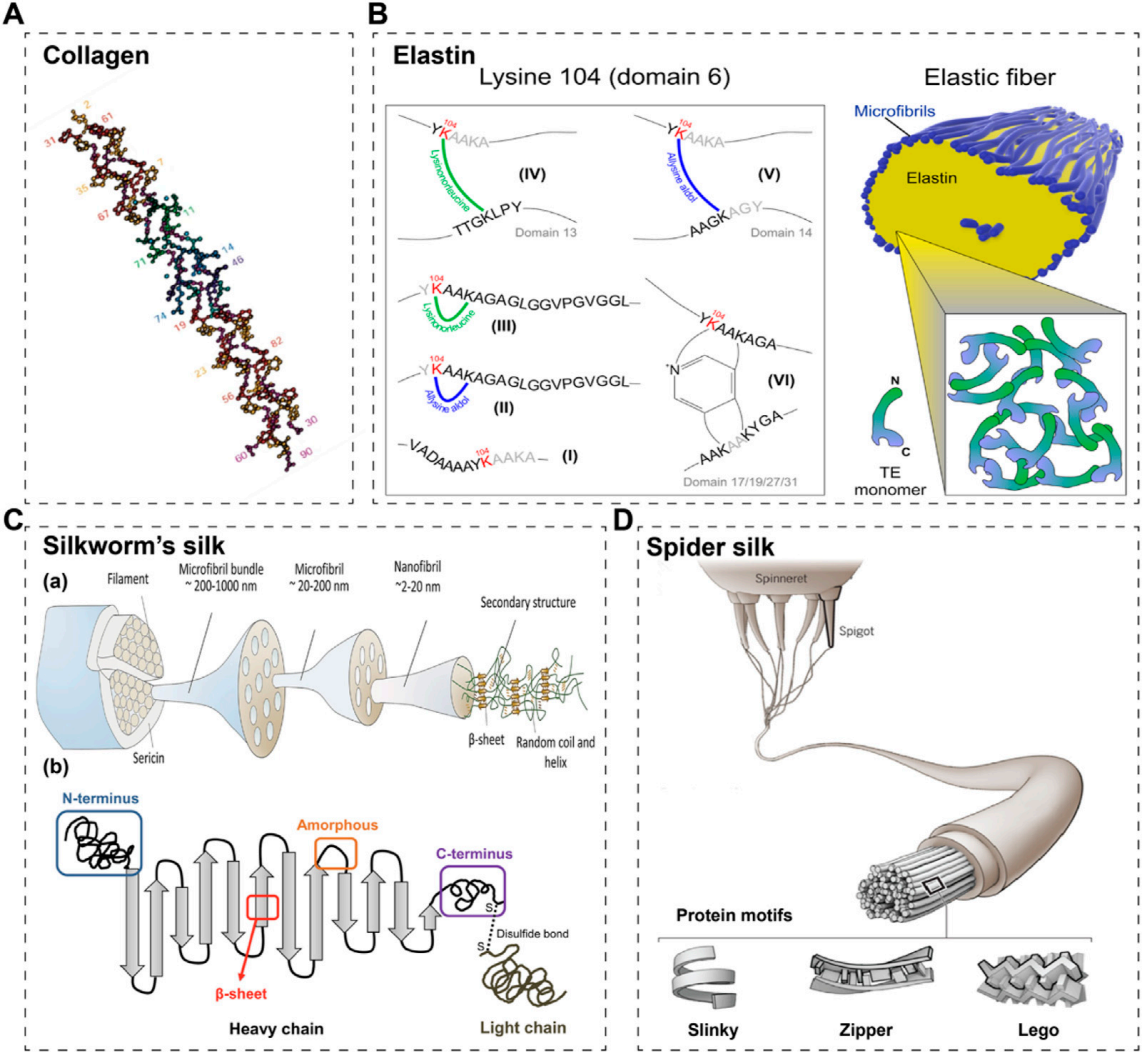
Schematic structures of commonly used fibrous proteins in STE. **(A)** A schematic of collagen’s triple helix structure (Kramer et al., 1999). **(B)** A schematic of elastin and its crosslinking sites (Schmelzer et al., 2020). **(C)** A schematic of silkworm’s silk (a) (Zhang and Fan, 2021) and its 2D cartoon (b) (Wongpinyochit et al., 2018). **(D)** The schematic of spider silk (Pennisi and Service, 2017).

Collagen is one of the main components of extracellular matrix (ECM) in skin and includes diverse members. These collagenous family members share some common characteristics that distinguish them from other proteins (Gelse et al., 2003; Ricard-Blum et al., 2005). They individually present rod-like structures and consist of three collagen α-chains folded into a triple-helix structure (Kramer et al., 1999) (Figure 1A). Besides, each collagen α-chain consists of the Gly-X-Y motif repeats. Any amino acid residue may occupy the X and the Y positions of the Gly-X-Y triplets. However, the Gly residues at every third position are mandatory to allow the folding of the α-chains into a compact triple-helix conformation (Shoulders and Raines, 2009). The proportion of collagen in ECM directly determines the mechanical properties, appearance and functions of skin (Lovell et al., 1987; Tzaphlidou and Zervakis, 2004; Rong et al., 2008; Kahan et al., 2009; Zorina et al., 2022; Liu H. et al., 2023; Voller and Rahman, 2023). Due to its excellent bio-functions, biocompatibility and biodegradability, collagens have been widely used in STE. Most of the commercial collagens are extracted from animal tissues. About three decades ago, researchers started to use recombinant DNA technology and cell lines to obtain various types of recombinant collagens (Schnieke et al., 1987; Olsen et al., 1991; Specks et al., 1992; Fertala et al., 1994); nowadays, many expression systems have been develop for recombinant collagens (Bulleid et al., 2000; Fertala, 2020). Basically, recombinant type I and III have mostly been used in STE and will be introduced in the following parts.

#### 2.1.1 Recombinant collagen type I

Type I collagen serves as the primary component of the skin extracellular matrix (ECM), constituting 80%–85% of the dermal ECM. It plays a vital role in maintaining the mechanical integrity and appearance of the skin, while also providing sites for cell adhesion. Various expression systems can be adopted for the production of recombinant type I collagen, including *E. coli* systems, mammalian cell systems, yeasts, and insect cells. Mammalian cell systems and insect cells have the capability to express correctly modified and thermostable type I collagen with a triple-helix structure (Fertala, 2020). However, due to the high cost and limitations on scalable production of cultured cell systems, *E. coli* and yeast systems are commonly used for collagen production. Nevertheless, these systems require the co-expression of prolyl 4-hydroxylase (P4H) subunits to facilitate the formation of collagen chains into a triple-helical conformation and ensure thermostability (Myllyharju et al., 2000). Additionally, proteolytic enzymes are employed to extract collagens from crude materials, albeit this process inevitably leads to the destruction of procollagen peptides and telopeptides. Consequently, due to the lack of intact telopeptides, the isolated recombinant type I collagens are unable to form proper fibrillar assemblies similar to those in the native tissue matrix (Prockop and Fertala, 1998; Steplewski et al., 2007; Shayegan et al., 2016). Thus, the structural and mechanical properties of recombinant collagen are inferior to naturally extracted collagen fibrils.

#### 2.1.2 Recombinant collagen type III

Collagen type III is another crucial component of the skin matrix, constituting approximately 8%–11% of the dermal extracellular matrix (ECM). Recent research has demonstrated that during the aging process, the proportion of collagen type III in human skin gradually decreases (Lovell et al., 1987; Tzaphlidou and Zervakis, 2004). Additionally, studies have shown that collagen type III can significantly reduce scar formation and promote skin regeneration (Wang J. et al., 2022; Huang et al., 2022; Xiong et al., 2023), making it increasingly popular in skin repair applications. The expression systems for collagen type III are similar to those for collagen type I. Cultured cell systems, while effective, are associated with high costs and are not suitable for scalable production. Consequently, *E*. *coli* and yeasts remain the primary systems for scalable production. In China, Shanxi Jinbo Biopharmaceutical Ltd. Utilizes the *E. Coli* system to express collagen type III and has developed a filler comprising freeze-dried type III collagen fibrils. Another company in China, Bloomage Biotechnology Ltd., employs *Pichia pastoris* (yeast) as the expression system. Studies have indicated that recombinant human collagen synthesized by yeast closely resembles native collagen compared to that produced by *E. coli* systems; however, yeast systems can only yield homotrimers, making them more suitable for scalable production of collagen type III (homotrimer) (Vaughan et al., 1998; He et al., 2015). Similar to recombinant collagen type I, recombinant collagen type III also faces challenges in forming proper fibrillar assemblies due to the lack of intact telopeptides.

Table 2 summarizes crucial expression systems for the production of recombinant collagen type I and III.

**TABLE 2:** A summary of the expression systems for production of recombinant human collagen type I and III.

	Expression system	Transduced genes	Collagen types in skin	Refs
Mammalian cell	NIT 3T3, HT1080	*COL1A1*	I	Olsen et al. (1991), Geddis and Prockop (1993)
Prokaryote	*Escherichia coli* (*E. coli*)	*COL1A1, PH4A/B*	I	Buechter et al. (2003), Rutschmann et al. (2014), Liu et al. (2023b)
	*E. coli*	*COL3A1, PH4A/B*	III	Rutschmann et al. (2014), Shi et al. (2017a)
Yeast cells	*Pichia pastoris*	*COL1A1, PH4A/B*	I	Vuorela et al. (1999), Myllyharju et al. (2000), Báez et al. (2005)
	*Pichia pastoris*	*COL3A1, PH4A/B*	III	He et al. (2015), Xu et al. (2015), Shi et al. (2017b), Xin et al. (2020), Fang et al. (2023)
Insect cells	Spodoptera frugiperda (Sf9 cells)	*COL3A1*	I, III	Lamberg et al. (1996), Myllyharju et al. (1997)

### 2.2 Recombinant elastin-like peptides/proteins

Elastin is an important extracellular matrix protein in animals, mainly composed of elastic fibres, which provide elasticity and flexibility to a wide range of tissues (e.g., blood vessels, ligaments, lungs and skin) (Almine et al., 2010; Vindin et al., 2019). Natural elastin, one of the most stable proteins in the body, is a highly insoluble matrix protein that forms fibre networks due to interactions between hydrophobic fragments (Xiao et al., 2021). It is rich in hydrophobic amino acids and has repetitive tetra-, penta- and hexa-peptide sequences including Val-Pro-Gly-Gly (VPGG), Val-Pro-Gly-Val-Gly (VPGVG) and Ala-Pro-Gly-Val-Gly-Val (APGVGV), respectively (Urry et al., 2002; Karle and Urry, 2005; Schmelzer et al., 2020) (Figure 1B). Elastin plays an important role in the regulation of a variety of cellular functions, including the promotion of cell adhesion, proliferation, differentiation, chemotaxis and migration (Hinek et al., 2008). Based on these characteristics, elastin is considered an ideal raw material for the preparation of medical materials. However, elastin is insoluble in water, and it is more difficult to study elastin, which limits its uses. Besides, elastin extracted from natural animal tissues has a high immunogenicity and at the same time a relatively homogenous sequence. Chemically synthesized elastomeric peptides are less capable of forming structurally regular macromolecular materials and are more expensive to prepare. As a result, recombinant synthesis methods are considered.

In 1973 Dan Urry and his colleagues discovered that natural elastin contains a large number of VPGVG repeated amino acid sequences (Urry et al., 1991). Based on this discovery, the researchers synthesized a recombinant protein polymer, elastin-like polypeptide (ELP), using gene synthesis based on the repetitive amino acid sequence of the hydrophobic region of elastin (Castiglione Morelli et al., 1993). The commonly designed ELP is a pentapeptide repeat oligomer with a pentapeptide repeat sequence of (Val-Pro-Gly-X-Gly) VPGXG, where X can be any amino acid other than L-proline. ELP has peptides with amino acid sequences similar to elastin that mimic elastin properties to some extent (Meyer and Chilkoti, 1999a). As different amino acids possess different structures and functions, ELP can exhibit different biological functions with changes in X.

*Escherichia coli* (*E. coli*) expression system is most frequently utilized to produce ELP (McPherson et al., 1996). The sequence and length of ELPs can be designed by genetic engineering technology, which can make the molecular weight, composition and dispersion completely precise and controllable. The elastin prepared by this method has the advantages of low cost, high expression capacity and easy to produce in large scale (Bataille et al., 2015). Additionally, ELP has exceptional temperature responsiveness; it exhibits reversible phase transition behavior with temperature change (Cao et al., 2019; Santos et al., 2019). When the temperature is below its phase transition temperature (Tt), ELP dissolves in solution as a monomer. At temperatures higher than Tt, ELP is in an insoluble aggregated state (Shi et al., 2014; Zhao et al., 2016). Tt can be controlled by varying the number of X and pentapeptide sequence repeats in the ELP (Meyer and Chilkoti, 2004). Table 3 summarizes common recombinant elastin-like peptides/proteins.

**TABLE 3:** A summary of the common recombinant elastin-like peptides/proteins.

Expression system	Product sequence	Ref
*Escherichia coli* (*E. coli*)	(VPGVG)120 (GY)7	Jenkins et al. (2021)
	(VPGVG)2VPGCG (VPGVG)2	Monfort and Koria (2017)
	(GVGVP)4 (GYGVP) (GVGVP)3	Roberts et al. (2018)
	VPGVG (VPAGVG)6	Ciofani et al. (2014)
	VPAVG	Li et al. (2023a)
	APAAAAAAKAAAKAAQF GLVPGVG VAPGVG VAPGVG LAPGVG VAPGVG VAPGI; PGAPAA GLVPGVG VAPGVG VAPGVG LAPGVG VAPGVG VAPGIG	Celebi et al. (2012)
	[(A/G) GVPG]80; (SGVPG)80	Weitzhandler et al. (2017)
	V4L4G1-9	van Eldijk et al. (2012)
	(VPGIG)20; (VPGIG)40; (VPGIG)60	Bataille et al. (2016)

### 2.3 Recombinant silk proteins

Silk fibroin and spidroin are two types of silk proteins extensively utilized in skin tissue engineering (STE) (Kundu et al., 2013; Chouhan and Mandal, 2020; Gholipourmalekabadi et al., 2020; Salehi et al., 2020). Natural silk fibroin is produced by silkworms, particularly *Bombyx mori*, while spidroin is secreted by spiders. However, current industrial extraction processes for silk fibroin can still impact environmental sustainability and the final quality of the product. Additionally, the domestication of spiders for large-scale silk production is challenging due to their territorial nature. To address these challenges, scientists have turned to recombinant DNA technology and microbial expression systems to produce silk proteins with improved sustainability and quality control (Aigner et al., 2018). This approach offers promising alternatives to traditional extraction methods, enabling more environmentally friendly and controlled production processes.

#### 2.3.1 Recombinant silk fibroin

Silk fibroin, extracted from silk fibers, is widely utilized in skin tissue engineering (STE) owing to its outstanding biocompatibility, mechanical properties, and processability. At the molecular level, fibroin comprises three components: the heavy chain, the light chain, and the glycoprotein, with the heavy chain (H-ch) being the most crucial (Suzuki, 2016; Wongpinyochit et al., 2018; Zhang and Fan, 2021) (Figure 1C). The H-ch features a highly repetitive sequence consisting predominantly of glycine (43%), alanine (30%), and serine (12%). These amino acids are arranged in 12 crystalline domains, primarily characterized by Gly-X repeats, where X is typically Ala (65%), Ser (23%), or Tyr (9%). The recurrent amino acid motif Gly-Ala-Gly-Ala-Gly-Ser has been directly detected using solid-state nuclear magnetic resonance (NMR) spectroscopy. Furthermore, the 12 crystalline domains are interspersed by 11 very similar spacer sequences, known as amorphous motifs (F1/F4/F8, amorphous F-motif) (Zhou et al., 2001; He et al., 2012).

Despite the well-domesticated nature of silkworms and the complexity of silk fibroin structures, there have been limited studies on the heterologous production of silk fibroin. *Escherichia coli* is commonly employed for recombinant silk fibroin expression. However, the high frequency of homologous recombination among tandem repeat sequences renders the *E. coli* expression system unstable for silk fibroin production (Rosano and Ceccarelli, 2014b; Macek et al., 2019). Additionally, meeting the high demand for tRNAs corresponding to glycine and alanine, the two most abundant amino acids in the H-ch, poses a challenge, resulting in the production of small fibroin proteins (Xia et al., 2010). In attempts to address the issue caused by the high repeating frequency, researchers have increased the number of amorphous motifs, albeit this has led to more α-helix domains and poorer mechanical properties (Wu et al., 2017). Consequently, scientists have utilized *E. coli* systems to separately express the crystalline region and the amorphous region to investigate their biomedical functions individually (Yang et al., 2016). Table 4 summarizes the expression systems of recombinant silk fibroin and the recombinant silk fibroin products.

**TABLE 4:** A summary of the expression systems of recombinant silk fibroin and the recombinant silk fibroin products.

Expression system	Recombinant product	Refs
*Escherichia Coli (E. coli)*	H-ch repetitive domain: [GAGAGS]16-tag; [GAGAGV]16-tag [GAGAGA]16-tag	Wang et al. (2009)
*E. coli*	H-ch amorphous domain F1; F4; F8	Yin et al. (2016)
*E. coli*	H-ch repetitive domain and amorphous domain: [GAGAGS]16-F1-tag [GAGAGS]16-F4-tag [GAGAGS]16-F8-tag	Wu et al. (2017)
*Pichia pastoris*	H-ch repetitive domain	Manoharan et al. (2022)

#### 2.3.2 Recombinant spidroin (spider silk)

Spider silks have been utilized for centuries in hemostasis and wound treatment and continue to be prominent in biomedical science today (Salehi et al., 2020; Bakhshandeh et al., 2021). Spiders have the ability to produce approximately seven different types of silk using various silk glands and spinnerets (Pennisi and Service, 2017) (Figure 1D). These silks possess distinct amino acid sequences and functions (Poza et al., 2002; Hu et al., 2006; Hardy et al., 2008). Among them, major ampullate (MA) silk, also known as dragline silk, has been extensively studied (Work, 1985; Knight and Vollrath, 2001; Lawrence et al., 2004). MA silk is composed of two highly conserved spidroins: the proline-free major ampullate spidroin (MaSp) one and the proline-rich MaSp 2 (Hu et al., 2006). Typically, MaSp one is hydrophobic, while MaSp two is hydrophilic (Huemmerich et al., 2004); moreover, the hydropathy of these two spidroins may vary among spider species. Colline et al. discovered a new spidroin, MaSp 3, which differs from the typical two spidroins by lacking polyalanine and glycine-proline-glycine domains, while containing larger and more polar amino acids in its repeat motifs (Collin et al., 2018).

Due to the challenges associated with harvesting natural spidroins, recombinant production of spidroins has received more attention than that of silk fibroin. The most commonly used recombinant spidroins are based on DNA sequences from *Nephila clavipes* or *Araneus diadematus* (Humenik et al., 2014; Aparecido dos Santos-Pinto et al., 2018). Various expression systems can be employed, including *Escherichia coli*, Pichia pastoris, mammalian cells, and insect cells (Vendrely and Scheibel, 2007; Heidebrecht and Scheibel, 2013). Among these, *E. coli* is the most prevalent due to its fast growth and ease of transformation (Rosano and Ceccarelli, 2014b). However, the discrepancy in codon usage between spiders and *E. coli* remains a significant concern. Additionally, as observed in silk fibroin recombinant production, bacteria often eliminate repetitive sequences through homologous recombination, resulting in the production of small spidroins (Arcidiacono et al., 1998). To address these challenges, scientists must optimize genetic information and bacteria codon usage (Heidebrecht et al., 2015). Through this strategy, it becomes feasible to produce sufficient and tailored spidroins with varying lengths or amino acid sequences (Heidebrecht and Scheibel, 2013; Heidebrecht et al., 2015). Table 5 summarizes the expression systems of recombinant spidroins and the recombinant products.

**TABLE 5:** A summary of the expression systems and gene sources for production of recombinant spidroins.

	Expression system	Gene source	Product	Refs
Mammalian cell	Hamster kidney cells	Major ampullate *Nephila clavipes*	Masp 1 Masp 2	Grip et al. (2006)
Prokaryote	*Escherichia coli (E. coli)*	Major ampullate *Nephila clavipes*	Masp 1	Fahnestock and Irwin (1997), Xia et al. (2010)
	*E. coli*	Major ampullate *Argiope aurantia*	Masp 2	Brooks et al. (2008)
Eukaryote	*Pichia pastoris*	Major ampullate *Nephila clavipes*	Masp 1	Fahnestock and Bedzyk (1997)
Insect cell	Spodoptera frugiperda (sf9 cell)	Major ampullate *Araneus ventricosus*	ASP	Liu et al. (2011)

### 2.4 Recombinant chimeric proteins

Another notable advantage of recombinant production is its facilitation of obtaining chimeric biomaterials, allowing scientists to create proteins with complementary properties, thereby leading to the development of new or optimized functions (Hayashi et al., 2001; Plowright et al., 2016; Addi et al., 2017; Dinjaski et al., 2017; Humenik et al., 2018). The elastin-silk protein serves as a prominent example in this realm (Dinjaski and Kaplan, 2016; Chambre et al., 2020). Researchers could combine motifs from fibrous protein to engineer recombinant proteins with enhanced mechanical properties and temperature-responsive abilities (Bessa et al., 2010; Gomes et al., 2011; Muiznieks and Keeley, 2016; Petitdemange et al., 2017; Rim et al., 2017; Isaacson et al., 2018; Le Fer et al., 2019; Patkar et al., 2024). Moreover, short cell adhesive peptides can be incorporated into recombinant fibrous proteins to promote cell adhesion (Tanaka and Asakura, 2009; Kim et al., 2010; Wang et al., 2017). Consequently, researchers can leverage recombinant technology to engineer innovative biomaterials tailored to different applications by integrating the physical, chemical or biological advantages of various proteins.

## 3 Applications in STE

In terms of skin tissue engineering, natural fibrous proteins (collagens, silk proteins, elastin, etc.) have been widely used in constructing 3D scaffolds, involving different forms, such as hydrogels, fibers, foams, etc. They can provide mechanical support and proper microenvironment for cell migration and adhesion. Traditionally, these fibrous proteins are extracted from animal tissues or products, which might contain infectious pathogens, threatening the health of human beings (Li Z.-H. et al., 2013; Bakhshandeh et al., 2021; Akdag et al., 2023; Cao et al., 2024a; De Giorgio et al., 2024; Gardeazabal and Izeta, 2024; Karahisar Turan et al., 2024; La Monica et al., 2024; Santos et al., 2024). Herein, recombinant DNA technology offers appealing alternatives to natural fibrous proteins, rendering them more accessible for scientific and medical applications. Additionally, the exceptional modular designing ability and bio-safety of these recombinant biomaterials have captured the attention of scientists and medical professionals alike. Consequently, recombinant biomaterials are progressively gaining prominence in biomedical applications. This trend is also evident in skin tissue engineering (STE), where scientists have begun integrating recombinant biomaterials with other advanced technologies such as 3D printing and organoid culturing to develop innovative skin scaffolds or equivalents for skin regeneration. In this section, we will focus on the principal representative applications of recombinant fibrous proteins (including collagen, elastin, silk fibroin, spidroin, and chimeric fibrous proteins) and the chimeric fibrous proteins in STE, aiming to provide guidance to those interested in exploring this field further.

### 3.1 Recombinant collagen in STE

In skin tissue engineering (STE), recombinant human type I and III collagens stand out as two of the most commonly utilized collagens, given their significance as primary components of the human skin extracellular matrix (ECM). Recombinant collagens have been employed alone or combined with other biomaterials, such as chitosan and hyaluronic acid, to create a variety of scaffolds for skin regeneration (Deng et al., 2018; Cao et al., 2020; Cheng et al., 2020; Yang et al., 2022; Liu T. et al., 2023; Kang et al., 2023; Xiong et al., 2023).

**FIGURE 2:**
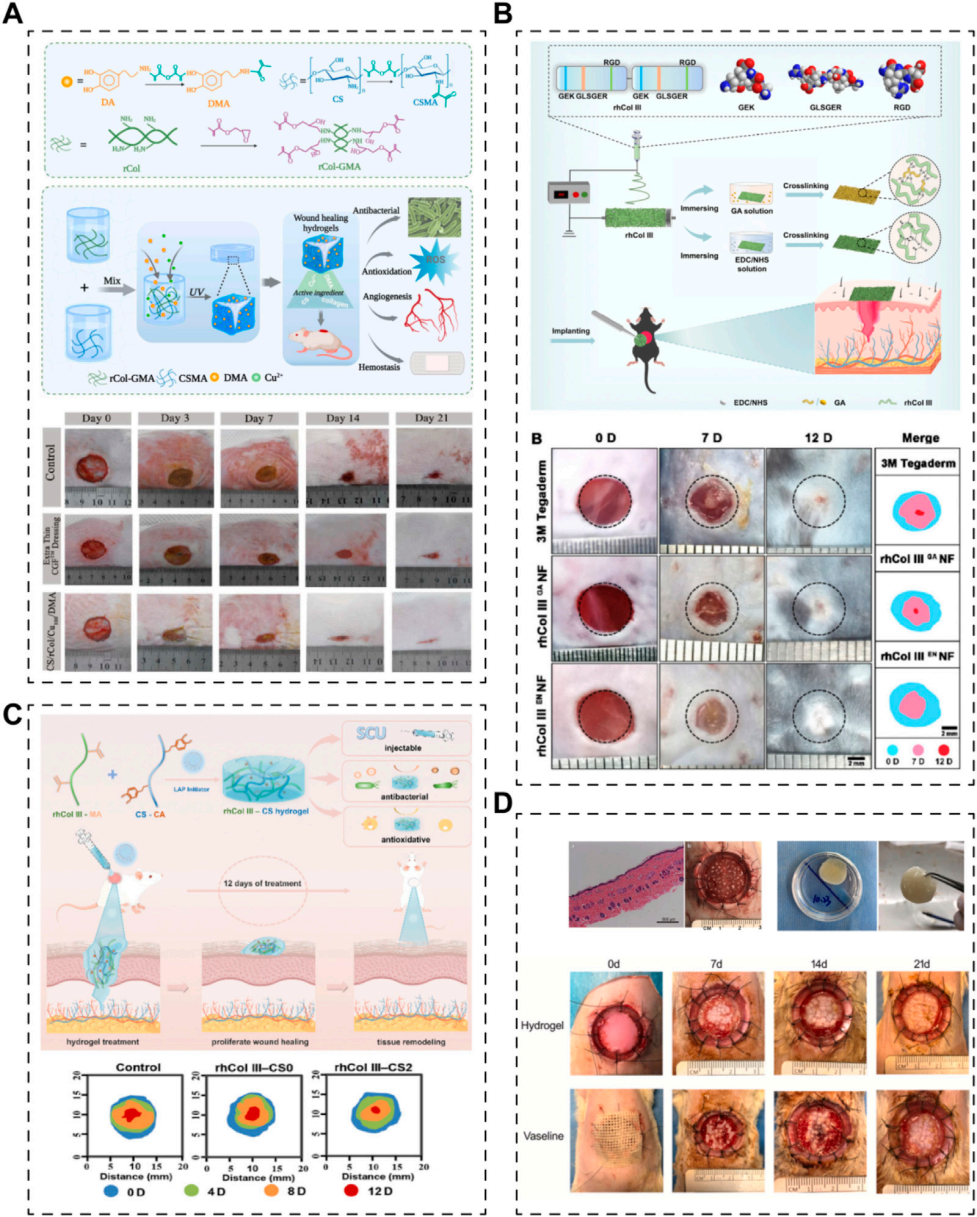
Various skin scaffolds containing recombinant human collagens. **(A)** A hydrogel based on recombinant type I collagen (rCol)/chitosan (CS) scaffold to accelerate full-thickness healing of skin wounds (Kang et al., 2023). **(B)** Electrospun nanofibrous membranes of recombinant human collagen type III (rhCol III) promote cutaneous wound healing (Dong et al., 2023). **(C)** Injectable hydrogels of recombinant human collagen type III (rhCol III) and chitosan (CS) with antibacterial and antioxidative activities for wound healing (Xiong et al., 2023). **(D)** A recombinant human collagen hydrogel as minced split-thickness skin graft overlay to promote full-thickness skin defect reconstruction (Liu T. et al., 2023).

For instance, Kang et al. recently developed a double-network hydrogel scaffold for skin regeneration, incorporating recombinant human collagen type I (expressed in *E. coli*) (Figure 2A) (Kang et al., 2023). In their study, recombinant human collagen type I and chitosan were glycidyl methacrylated and methacrylated, respectively, to form an ultraviolet-induced crosslink network. Additionally, a physical crosslinking network was established between Cu^2+^ and the catechol group of dopamine methacrylamide (DMA). The scaffold exhibited antibacterial, antioxidative, angiogenic, and hemostatic properties. However, the specific role of recombinant collagen in skin regeneration was not separately investigated. In another study, scientists utilized green electrospinning technology to create a nanofibrous scaffold incorporating integrin receptor-incorporated recombinant human collagen type III (Figure 2B) (Dong et al., 2023). This scaffold significantly accelerated wound closure and promoted the recovery of skin structures and appendages in a mouse model of full-thickness skin defects. Notably, the study compared the effects of different crosslinking systems (glutaraldehyde or EDC/NHS) on the nanofibrous scaffold, with the EDC/NHS system resulting in better physical properties. Xiong et al. developed an injectable hydrogel system containing methacrylated recombinant human collagen type III (expressed in *P. pastoris*) and chitosan (Figure 2C) (Xiong et al., 2023). The recombinant human collagen type III enhanced cell adhesion, migration, and proliferation, while chitosan provided antibacterial properties, collectively accelerating skin regeneration. Furthermore, this recombinant human collagen type III-based hydrogel could serve as an overlay for minced split-thickness skin grafts, promoting skin defect regeneration (Figure 2D) (Liu T. et al., 2023). The hydrogel facilitated angiogenesis and collagen deposition at the wound sites, leading to reduced scarring and improved appendage regeneration. These strategies, focusing solely on biomaterials without cells or growth factors, demonstrate the versatility of recombinant human collagens in various forms to meet the requirements for skin repair.

**FIGURE 3:**
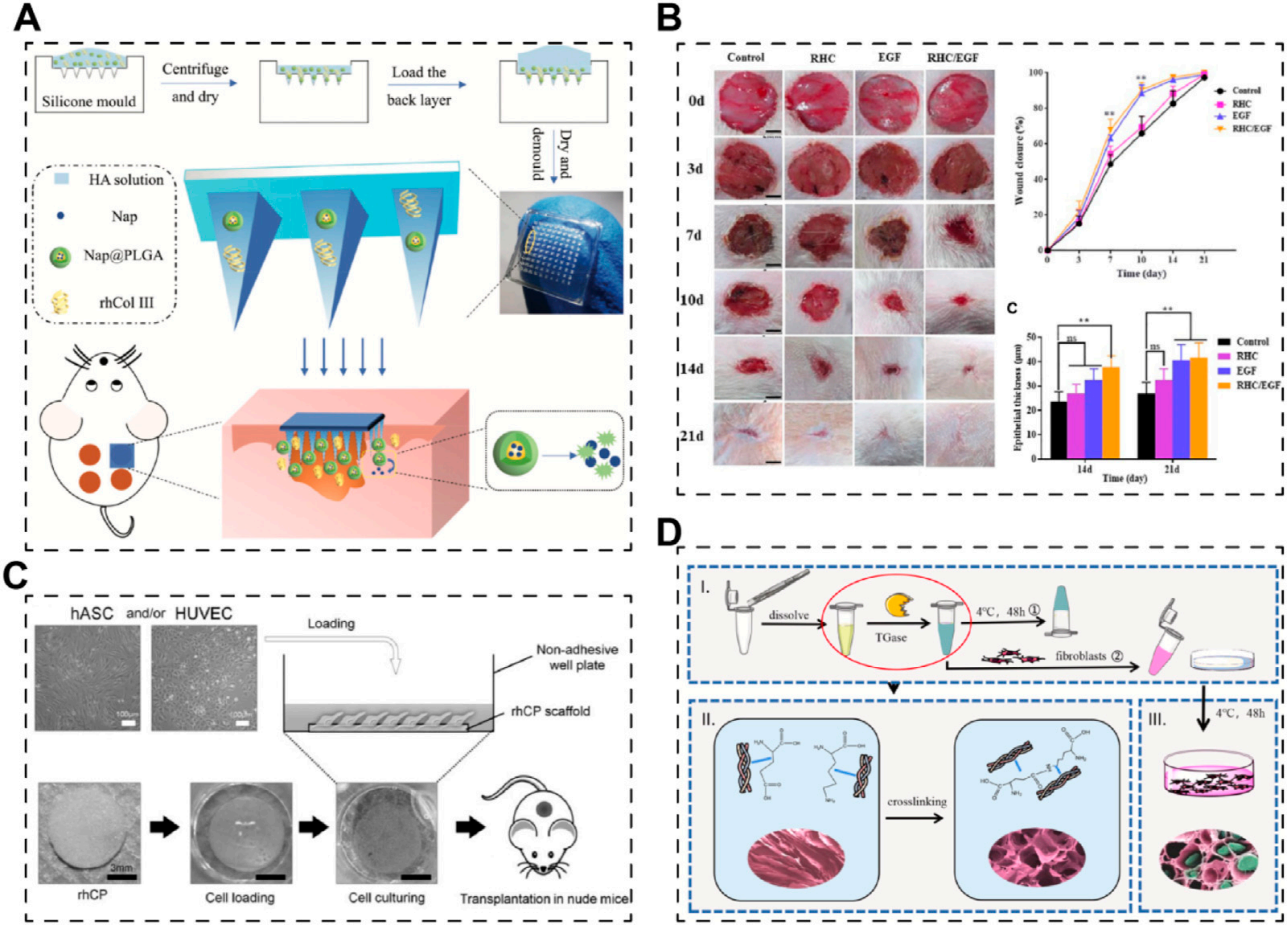
Skin scaffolds containing recombinant human collagens and drugs, growth factors or cells. **(A)** A dissolving microneedle system (hyaluronic acid, HA), encapsulating drug-loaded nanoparticles (nap@PLGA) and recombinant humanized collagen type III (rhCol III), for the treatment of chronic wound (Long et al., 2022). **(B)** Hybrid freeze-dried dressings (RHC/EGF) composed of epidermal frowth factor (EGF) and recombinant human collagen (RHC) enhance cutaneous wound healing in rats (Cheng et al., 2020). **(C)** A recombinant human collagen peptide (rhCP) bioscaffold with human adipose-derived stem cells (hACSs) or endothelial cells (Mashiko et al., 2018). **(D)** A tissue-engineered skin equivalent consists of recombinant human collagen hydrogel and fibroblasts (Guo et al., 2021).

On the other hand, drugs, growth factors, or even cells could also be incorporated into the scaffolds system to realize efficient skin regeneration. Long et al. established a dissolving hyaluronic acid microneedle system; recombinant human collagen type III (*E. coli* expression system) and naproxen (NSAID) acted as additives to the microneedle (Figure 3A) (Long et al., 2022). The results demonstrated that recombinant human collagen type III remarkably promoted cell migration and proliferation, while naproxen reduced inflammation level, together benefiting the recovery of chronic wounds. Besides, growth factors, such as epidermal growth factor (EGF) and basic fibroblast growth factor (bFGF), have been used with recombinant human collagen to augment the ability of scaffolds to promote skin regeneration (Figure 3B) (Guo et al., 2019; Cheng et al., 2020). These studies together proved that recombinant human collagen-based hydrogels could be delivery systems for growth factors. Further, Mashiko et al. used human adipose-derived stem cells (hADSCs)-loaded recombinant human collage type I peptide (yeast expression system) hydrogel to treat wound after radiotherapy (Figure 3C) (Mashiko et al., 2018). Another team from China established a tissue-engineered skin equivalent using recombinant human collagen (E.Coli expressing system) and fibroblasts to repair skin defects (Figure 3D) (Guo et al., 2021). Their research suggests that recombinant human collagen-based hydrogels act as biocompatible bio-carrier for cells and stimulate the growth factor secreting of the carried cells, indicating recombinant human collagens could be used in tissue engineering-based cell therapies.

Indeed, the aforementioned advanced studies collectively underscore the effectiveness of recombinant human collagens as alternatives to animal tissue-derived collagen. They show extraordinary biocompatibility and could promote cell adhesion as well as migration. Moreover, they hold promise for the development of tissue-engineered medical devices tailored for skin regeneration. Importantly, these devices can be designed with or without additional additives, including drugs, growth factors, and cells. This versatility not only enhances the potential applications of recombinant human collagens in skin tissue engineering but also opens up avenues for personalized and multifunctional approaches to address various skin regeneration challenges.

### 3.2 Recombinant elastin in STE

Elastin, another crucial fibrous protein in the skin extracellular matrix (ECM), plays a vital role in conferring elasticity and resilience to skin tissues. Recent studies have demonstrated the benefits of elastin in promoting the deposition of elastin fibers and collagen fibers at wound sites, thereby reducing scar formation (Daamen et al., 2008; Antonicelli et al., 2009; Xie et al., 2017; Khalili et al., 2019; Yamamoto and Kawamura, 2020). Consequently, recombinant human elastin has emerged as a promising candidate for skin tissue engineering (STE), either alone or as part of chimeric fibrous proteins fused with other biomaterial sequences or functional motifs.

**FIGURE 4:**
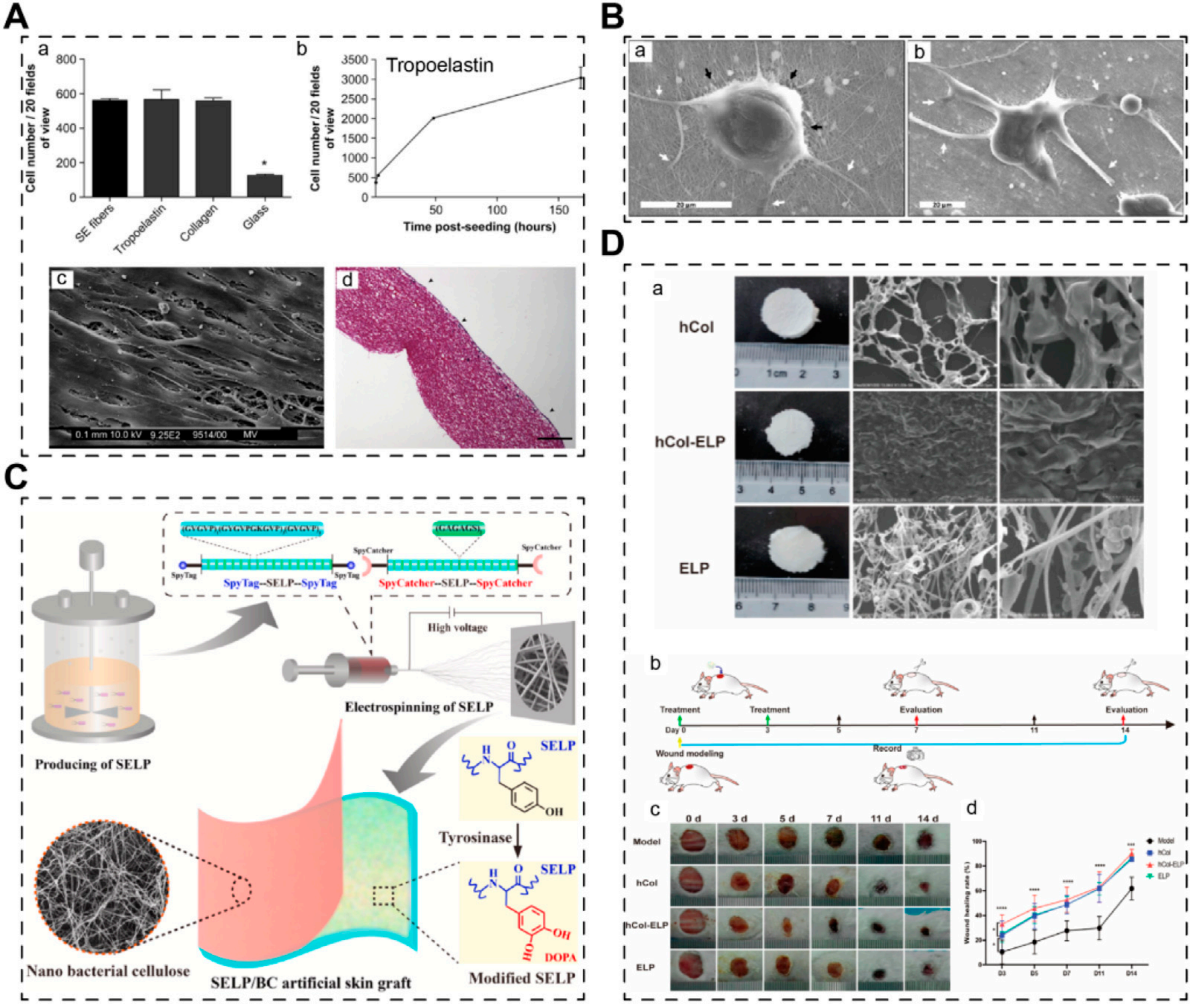
Skin scaffolds containing recombinant elastins or chimeric elastin-like proteins. **(A)** A 3D scaffold from recombinant tropoelastin promotes skin fibroblasts adhesion (Rnjak et al., 2009). **(B)** Fibroblasts attach and spread well on electrospun recombinant elastin scaffolds (Machado et al., 2013). **(C)** A electrospun skin substitute consists of recombinant elastin-silk-like protein (SELP) (Feng et al., 2024). **(D)** A scaffold consists of recombinant elastin-collagen polypeptide (hCol-ELP) promotes skin regeneration (Chen Y. et al., 2023).

Studies focusing on recombinant elastin alone are relatively limited in the context of STE. For example, Jelena Rnjak et al. developed crosslinked scaffolds based on recombinant tropoelastin (expressed in *E. coli*), the precursor of elastin in the human body (Figure 4A) (Rnjak et al., 2009). Their primary objective was to create a non-animal material-based skin substitute that outperforms collagen scaffolds in terms of *in vivo* contraction. They utilized electrospinning to fabricate highly elastic nanofibrous scaffolds with a Young’s modulus of 265 ± 17 kPa. These scaffolds exhibited excellent cytocompatibility with human skin fibroblasts, highlighting their potential for skin regeneration. Future investigations could explore the use of recombinant elastin to enhance the mechanical properties of skin grafts.

In many cases, elastin sequences are fused with other biomaterial sequences or functional motifs to create chimeric recombinant fibrous proteins, leveraging the tunability afforded by recombinant technology. For instance, Machado et al. engineered an elastin-silk fibroin biomaterial (expressed in *E. coli*), combining conservative motifs from both proteins to develop a material with the high tensile strength of silk fibroin and the elasticity of elastin) (Figure 4B) (Machado et al., 2013). Their electrospun scaffolds exhibited significantly improved mechanical properties (modulus of elasticity ∼126 MPa) and supported the adhesion and proliferation of human skin fibroblasts. Similarly, Feng’s team utilized an *E. coli* expression system to produce an elastin-silk-like protein with exceptional mechanical properties (Figure 4C) (Feng et al., 2024). By combining this recombinant elastin-silk layer with a nano bacterial cellulose layer, they fabricated a bilayer skin substitute with excellent mechanical strength and antibacterial properties. Furthermore, elastins have been fused with collagen to create novel artificial biomaterials. Chen’s team reported a recombinant human collagen-elastin protein using an *E. coli* expression system (Figure 4D) (Chen Y. et al., 2023). The incorporation of elastin sequences increased the stability of human collagen, resulting in stable membrane scaffolds conducive to efficient skin regeneration. Thus, elastin sequences hold promise for engineering mechanically robust recombinant biomaterials, either alone or in combination with other proteins, for applications in skin tissue engineering.

**FIGURE 5:**
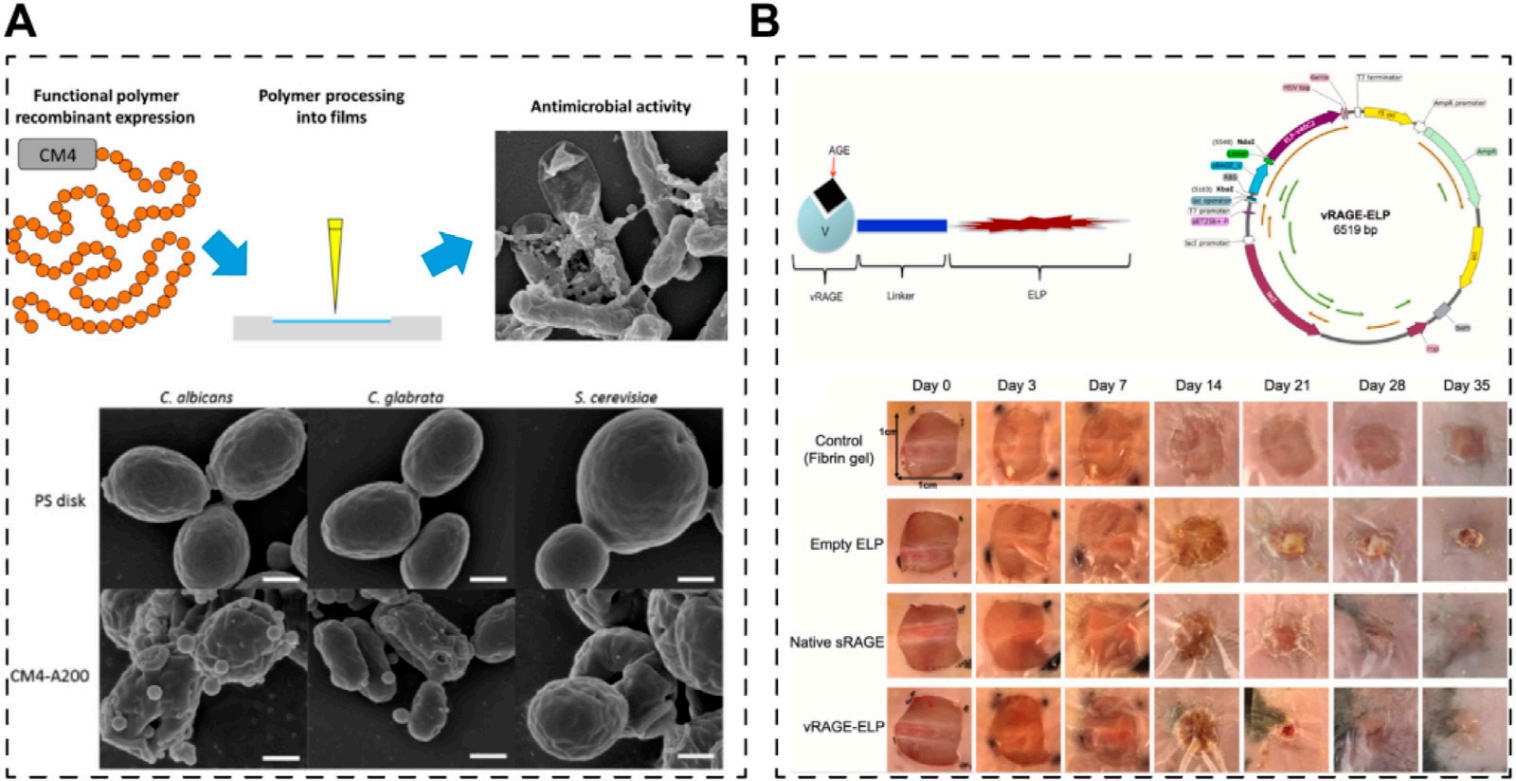
Skin scaffolds containing recombinant functional elastins. **(A)** An elastin-like recombinamer films with antimicrobial activity; CM4 is the antibacterial peptide fused with elastin peptide (da Costa et al., 2015). **(B)** A self-assembled elastin-like polypeptide fusion protein (vRAGE-ELP) coacervates as competitive inhibitors of advanced glycation end-products enhance diabetic wound healing (Kang et al., 2021).

Indeed, the fusion of elastins with other functional motifs has opened avenues for creating recombinant elastin-like proteins with diverse bioactive functions aimed at promoting skin regeneration or wound healing. These functions include enhancing cell adhesion, antibacterial properties, and managing diabetic wounds. In a study by Beste Kinikoglu et al., in 2011, a recombinant elastin fused with the RGD peptide (expressed in *E. coli*) exhibited significant cell-adhering capacity, making it suitable for the preparation of skin substitutes (Kinikoglu et al., 2011). Similarly, in 2015, a European team incorporated an antibacterial motif (ABP-CMP4 amino acid sequence) into elastin using an *E. coli* expression system (Figure 5A) (da Costa et al., 2015). The resulting recombinant elastin demonstrated remarkable antibacterial ability, showcasing its potential for use in tissue-engineered skin grafts, although further research in this area is warranted. Moreover, a team from the USA engineered a self-assembled elastin-like peptide with a functional motif capable of competing with advanced glycation end products (Figure 5B) (Kang et al., 2021). This innovative approach involved using fibrin gels to release the recombinant peptides, ultimately leading to the healing of diabetic wounds.

Together, recombinant elastin is also a great candidate for constructing skin scaffolds, in terms of biocompatibility and tissue-regeneration efficacy. The aforementioned studies underscore the versatility of elastin sequences, which can be fused with other functional motifs to serve as therapeutic additives in tissue-engineered scaffolds for treating various skin defects or chronic wounds. Notably, by incorporating elastin sequences, the mechanical properties of the chimeric protein biomaterial could be enhanced remarkably. Additionally, the anti-aging properties of elastin have garnered significant interest among scientists and dermatologists (Yan et al., 2022; Li J. N. et al., 2023; Yu-han et al., 2023). Although studies in this area primarily focus on repairing UV-damaged skin rather than addressing skin defects or wounds, they offer valuable insights into the potential applications of recombinant elastin in skin rejuvenation.

### 3.3 Recombinant silk proteins in STE

Silk fibroin and spidroins are both two kinds of insect-derived fibrous protein. As silkworms have been successfully domesticated for large-scale production of silk fibroin, little research has been done on the STE application of recombinant silk fibroin. Thus, in this part, the STE applications of recombinant silk fibroin and spidroin are summarized together.

Chitra Manoharan et al. bioengineered the heavy chain fragment of silk fibroin (rSF) using a *P. pastoris* expressing system; besides, cecropin B-functionalized silk fibroin heavy chain (rSFC) was also produced using the same expressing system (Manoharan et al., 2022). Actually, rSFC is a chimeric recombinant protein which has already been mentioned in this review. The authors compared their therapeutic efficacy on wound healing. It turns out that, rSFC is superior to rSF in wound healing, which proves that silk fibroin should be combined with other additives or biomaterials to realize efficient skin regeneration from another side. In addition, the rSF and rSFC were used once daily at a dosage of 50 μg for 3 days, without being processed into scaffolds or grafts in this study. Thus, it is not a conventional STE research, while it does involve bioengineered silk fibroin and evaluated their efficacy in wound repairing. Notably, transgenic silkworms or other creatures are also widely used for recombinant silk fibroin or chimeric recombinant silk fibroin production. However, we only discuss non-animal and non-plaint recombinant fibrous proteins here; more details could be found in the relevant references (Nagano et al., 2011; Kuwana et al., 2014; Yang et al., 2015; Wang et al., 2016; Lewis et al., 2021).

**FIGURE 6:**
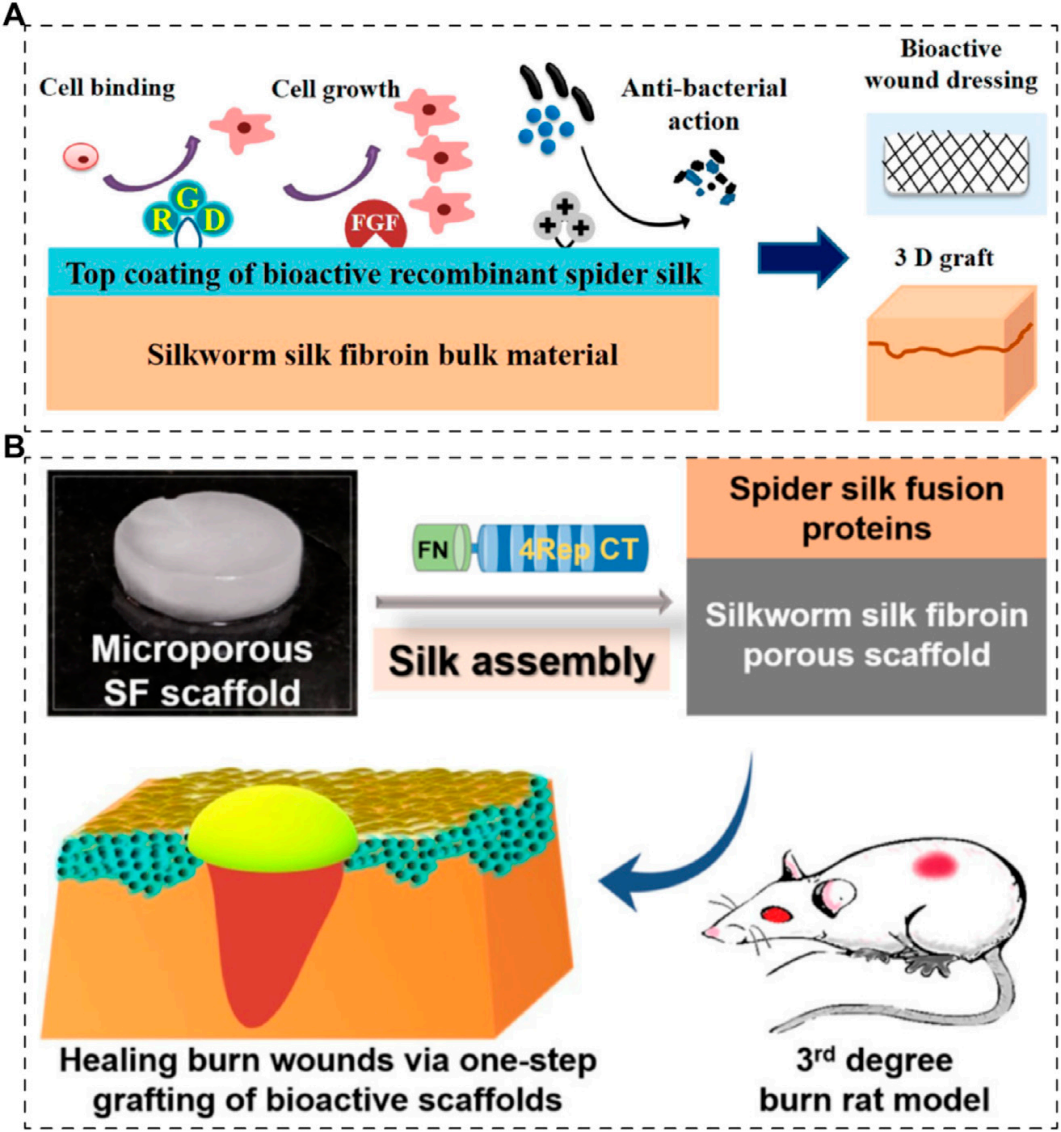
Skin scaffolds containing recombinant silk proteins. **(A)** Recombinant functionalized spider silk with silk fibroins matrices as potential bioactive 3D skin grafts (Chouhan et al., 2018). **(B)** Silk fibroin scaffolds functionalized with recombinant spider silk containing a fibronectin motif (spider silk fusion proteins) for full-thickness burn wounds regeneration (Chouhan et al., 2019).

For spidroins, they are usually functionalized, which means they are fused with functional motifs to be chimeric for applications. Dimple et al. prepared a 3D skin graft with silk fibroin matrices and functionalized recombinant spidroins (Figure 6A) (Chouhan et al., 2018). Silkworm-derived silk fibroin was fabricated into 3D scaffolds and then coated with recombinant spidroins (*E. coli* expressing system), fused with cell-binding motif from fibronectin, a growth factor (fibroblast growth factor) and an antimicrobial peptide respectively. They mentioned that recombinant spidroins could self-assemble to form stable coatings. However, the therapeutic efficacy was only investigated *in vitro*. Later, in 2019, the same team reported a research regarding recombinant spidroins containing a fibronectin motif (Figure 6B) (Chouhan et al., 2019). The 3D scaffolds were prepared in the same way with silkworm-derived silk fibroin as matrices and recombinant spidroin as a coating. Their results demonstrate that the recombinant spidroins containing a fibronectin motif are promising in treating burned wounds.

What’s more, some teams combined recombinant spidroins which are not chimeric with other biomaterials for applications. Wang et al. established a strategy of fabricating recombinant spidroins-based scaffolds (Zhu et al., 2020; Wang S. et al., 2022). Briefly, they blended recombinant spidroins (*E. coli* expressing system) and poly (L-lactide-co-ε-caprolactone) (PLCL); and then electrospinning was used for nanofibrous scaffolds constructing. The hemocompatibility and cytocompatibility of the scaffolds were proved to be excellent, while without *in vivo* investigation. Lian et al. prepared a recombinant spidroin (*E. coli* expressing system) nanofibrous membrane loaded with sodium hydrogen sulfide and endothelial progenitor cells for skin regeneration (Lian et al., 2021). The scaffold realized continuous H_2_S releasing and maintained the viability of the carried cells; they worked synergistically to regenerate the skin.

The aforementioned researches together suggest that no matter recombinant silk fibroins or spidroins should be fused with functional motifs for applications and the sequences of silk proteins basically serve as polymer chain backbones, providing stability. After being fused, they chimeric recombinant proteins could act as bioactive additives to other raw matrices and could also be utilized to establish delivery system for drugs or cells in STE. Taken together, recombinant fibrous protein (collagens, elastin and silk proteins) and the chimeric of them have already been widely studied in STE. Besides, the construction of tissue-engineered skin grafts is becoming more biomimetic, in terms of components, structures and functions. Looking into the future, more recombinant biomaterials and technologies will be used in STE to realize fast and efficient repair or regeneration; however, challenges and opportunities coexist for us.

## 4 Clinical applications

As many researches proved the therapeutic efficacy of recombinant fibrous proteins in skin repair, some pioneers in this field have already conducted clinical trials or launched commercial products, which further validate the translational potential of recombinant fibrous proteins in skin regeneration/rejuvenation. Specifically, recombinant collagen has become increasingly integral in the field of skin tissue engineering and repair/rejuvenation, both in clinical trials and commercial products. In clinical trials, researchers are exploring its use in developing advanced wound dressings and three-dimensional scaffolds aimed at enhancing wound healing and promoting skin regeneration. These scaffolds are typically engineered to closely mimic the natural composition and structure of native skin, leveraging recombinant human collagen types such as type I or type III (Yang et al., 2004; Ben et al., 2021; Cao et al., 2024b). The use of recombinant collagen in these applications aims to improve biocompatibility, support cell attachment, proliferation, and differentiation, and ultimately facilitate tissue integration and regeneration.

Commercially available products incorporating recombinant collagen in skin repair and rejuvenation include dermal fillers, which use collagen to restore volume and smooth out wrinkles and fine lines (Fertala, 2020). These fillers often use recombinant collagen type III to enhance skin elasticity and firmness. For example, the *Lyophilized Fiber of Recombinant Humanized Type III Collagen (Ayouth™)* from ***JINBO BIO-PHARMACEUTICAL CORPORATION LIMITED*** is used as dermal fillers for skin rejuvenation. Additionally, in the realm of anti-aging treatments, skin care products like creams and serums utilize recombinant collagen to improve skin texture, hydration, and overall appearance (Ma et al., 2022). These products capitalize on the ability of recombinant collagen to support the skin’s structural integrity and stimulate collagen production within the dermis, thereby reducing the signs of aging.

Overall, the clinical applications of recombinant collagen in skin tissue engineering and rejuvenation underscore its versatility and potential to advance therapeutic strategies for treating various dermatological conditions and enhancing aesthetic outcomes. Continued research and development in this field are expected to further broaden the scope and efficacy of recombinant collagen-based therapies for skin health and regeneration.

## 5 Future challenges and opportunities

So far, recombinant biomaterials have emerged as versatile tools with significant potential not only for STE, but also for other advancing healthcare, biotechnology, and beyond (Dandu and Ghandehari, 2007; Wang et al., 2013; Choi et al., 2018; Mandal et al., 2018; Garcia et al., 2023). As researchers continue to explore the capabilities of these engineered materials, several challenges and opportunities lie ahead. In this part, we discuss the future outlook for recombinant biomaterials, highlighting key challenges and opportunities for their development and applications.

### 5.1 Challenges

Skin is a highly complex organ composed of multiple layers, each with unique cellular compositions and functions. As tissue engineering advances, there’s a growing focus on recreating this complexity in engineered skin substitutes, involving mimicking the intricate interactions between keratinocytes, fibroblasts, immune cells, blood vessels, and nerves. Achieving spatial organization, cell-cell communication, and dynamic tissue remodeling in engineered constructs remains a formidable challenge. Furthermore, incorporating features such as hair follicles, sweat glands, and sebaceous glands adds another layer of complexity to tissue engineering efforts. Overcoming the complexity of future skin tissue engineering definitely requires innovative biomaterials that can provide spatial cues, biochemical signals, and mechanical support to guide cell behavior and tissue organization. With its modular designing nature, recombinant biomaterials will definitely take a place in future STE.

Despite advancements, concerns about immunogenicity and biocompatibility persist, particularly for long-term implantation or *in vivo* applications. Immune responses to implanted biomaterials may lead to rejection, inflammation, or adverse effects (Meek and Jansen, 2009; Kourtzelis et al., 2013; Shadrina et al., 2020; Doloff et al., 2021). Besides, the implantation of biomaterials not only affects the implantation sites but also the remote organs (Peng et al., 2023). Thus, understanding the immunological response to these recombinant biomaterials, especially over extended periods, is critical for ensuring their safety and efficacy in clinical applications. Besides, developing strategies to minimize immune reactions, enhance tissue integration, and modulate the host response is essential for improving the safety and efficacy of these biomaterials as well. For instance, protein engineering to minimize antigenicity or surface modifications to enhance biocompatibility, deserve further investigation (Lebre et al., 2016; Antmen et al., 2021; Ye et al., 2021; Dong et al., 2022; Rezaei et al., 2022; Backlund et al., 2023; Chen W. et al., 2023; Yousefpour et al., 2023).

Scaling up the production of recombinant biomaterials to meet clinical demands while maintaining cost-effectiveness presents challenges. Current production methods may face limitations in scalability, yield, and cost; developing scalable and cost-effective production processes, optimizing purification methods, and minimizing production-related impurities are necessary to ensure affordability and accessibility of these materials (Rice et al., 1993; Meyer and Chilkoti, 1999b; Mayer et al., 1999; Lojewska et al., 2016; Faravelli et al., 2021). Notably, the differences between the used expressing systems, such as post-translational modification and protein folding, profoundly affect the structure and functions of products as well, necessitating the systematic study to compare expressing systems in order to choose the optimized one for scale production (Fan et al., 2012; Gecchele et al., 2015).

Regulatory approval for clinical translation and commercialization of recombinant biomaterials involves navigating complex pathways governed by regulatory agencies such as the EMA, FDA or NMPA, which is challenging and time-consuming. Meeting stringent safety and efficacy standards, conducting comprehensive preclinical and clinical studies, and demonstrating long-term safety profiles are critical steps in the regulatory approval process. Furthermore, ensuring compliance with quality assurance and quality control standards throughout the manufacturing process is essential for obtaining regulatory clearance and market approval. Thus, collaborations between academia, industry, and regulatory agencies are essential for streamlining the regulatory process and accelerating the translation of promising technologies into clinical practice and commercial products.

Last but not least, the ethical considerations surrounding recombinant fibrous proteins are multifaceted, involving genetic modification, the use of animal models, and the potential impact on human health and the environment, as well as issues related to commercialization and accessibility. Genetic engineering raises concerns about unforeseen ecological consequences and the risks associated with gene editing techniques like CRISPR-Cas9. When evaluating the therapeutic efficacy of recombinant fibrous proteins, animal models are necessarily needed. However, the use of animal models necessitates careful attention to animal welfare and the exploration of alternative testing methods. Additionally, rigorous safety testing is essential to address potential health risks to humans, while environmental assessments are crucial to prevent accidental release and ecological disruption. The commercialization of these therapies also presents ethical challenges, including ensuring equitable access to treatments and addressing the implications of intellectual property rights on innovation and accessibility. Engaging stakeholders, including patients, policymakers, and the public, in ethical discussions is essential. Establishing clear ethical guidelines, ensuring informed consent, and promoting responsible stewardship of biological resources are crucial for navigating these complex ethical considerations.

### 5.2 Opportunities

Where there are challenges, there are opportunities as well. Due to the possibility of customizable and modular designing, recombinant biomaterials offer opportunities for personalized medicine by enabling tailored therapies based on individual patient characteristics. Not only for skin scaffolds and implants but also drug delivery systems, they can be customized and designed to match patient-specific needs, improving treatment outcomes and patient satisfaction. Specifically, with recombinant technology, multifunctional biomaterials can be engineered to deliver multiple therapeutic agents simultaneously, addressing various aspects of the wound healing process. For example, the recombinant biomaterials could include multiple functional motifs, such as growth factors, antimicrobial peptides, and extracellular matrix components, together to promote tissue regeneration while preventing infection. Furthermore, stimuli-responsive motifs could also be incorporated as well to dynamically release therapeutic agents in response to specific cues within the wound microenvironment, optimizing treatment efficacy. By tailoring the composition, structure, and properties of these multifunctional biomaterials, clinicians can develop personalized treatment strategies for complex wound conditions, such as diabetic foot ulcers, burn injuries, and chronic wounds associated with vascular or immune dysfunction.

Biomaterials can also be engineered to modulate immune responses and create a pro-regenerative environment within wounds (Lebre et al., 2016; Antmen et al., 2021; Ye et al., 2021; Dong et al., 2022; Rezaei et al., 2022; Backlund et al., 2023; Chen W. et al., 2023; Yousefpour et al., 2023). For example, recombinant biomaterials could be chimeric, which means they can be fused with immunomodulatory cytokines or mesenchymal stem cell-recruiting peptides to promote tissue regeneration while suppressing inflammation. Additionally, recombinant biomaterials functionalized with immune cell-recruiting peptides can enhance the recruitment of endogenous immune cells to the wound site, accelerating the healing process. Thus, by harnessing the body’s immune system and the recombinant technology in this way, clinicians can improve outcomes in conditions such as chronic wounds, autoimmune skin diseases, and tissue rejection following transplantation.

Finally, recombinant biomaterials can be integrated with other advanced engineering technologies, like 3D bioprinting and organ-on-a-chip. 3D bioprinting offers unprecedented control over the fabrication of skin constructs, allowing precise placement of cells and biomaterials to mimic the complex architecture of native skin (Kim et al., 2018; Patel et al., 2021; Shin et al., 2021; de Souza et al., 2023). For instance, researchers can bioprint skin grafts containing vascular networks, which are crucial for graft survival and integration. By incorporating recombinant biomaterials, patient-derived cells and disease-specific features into these engineered skin models, scientists can create personalized platforms for drug screening and disease modeling. Furthermore, recombinant biomaterials can be combined with organ-on-a-chip systems, to replicate the physiological microenvironment of skin tissue and enable researchers to study disease mechanisms and test potential therapeutics in a more accurate and clinically relevant context.

Together, challenges coexist with opportunities. Although there still are some major concerns waiting to be addressed, the future of recombinant biomaterials must be prosperous. Due to the limited natural sources and environmental pollution, recombinant biomaterials will greatly help human beings not only in healthcare but also in many aspects of our daily life. Thus, scientists, doctors and policymakers should work together to make breakthrough in this field.

## 6 Conclusion

Fibrous proteins like collagens, elastin, and silk proteins have long been integral to skin tissue engineering (STE). However, concerns such as cross-infection risks, extraction pollution, and batch-to-batch variations have spurred the search for alternatives. With advancements in recombinant DNA and fermentation technologies, recombinant fibrous collagens have emerged as promising solutions in STE. This review introduces recombinant human collagen types I and III, as well as recombinant elastin and silk proteins (silk fibroin and spidroin), along with their chimeric derivatives. We provide an overview of commonly used expression systems and the fundamental properties of these recombinant fibrous proteins. Subsequently, we analyze their diverse applications in STE. We anticipate that artificial skin grafts and substitutes will become increasingly biomimetic with the integration of recombinant biomaterials, enhancing their resemblance to natural skin in terms of composition, structure, and functionality. Moreover, we address the challenges and opportunities in this field, emphasizing the need for collaborative efforts among scientists, engineers, doctors, and policymakers to overcome technical, manufacturing, and commercialization obstacles. Despite existing challenges, we maintain a strong belief in the potential of recombinant biomaterials to thrive in personalized medicine, cross-disciplinary healthcare technologies, and everyday life.

## References

[B1] Addi C. Murschel F. De Crescenzo G. (2017). Design and use of chimeric proteins containing a collagen-binding domain for wound healing and bone regeneration. Tissue Eng. Part B-Reviews 23 (2), 163–182. 10.1089/ten.teb.2016.0280 27824290

[B2] Aigner T. B. DeSimone E. Scheibel T. (2018). Biomedical applications of recombinant silk-based materials. Adv. Mater. 30 (19), e1704636. 10.1002/adma.201704636 29436028

[B3] Akdag Z. Ulag S. Kalaskar D. M. Duta L. Gunduz O. (2023). Advanced applications of silk-based hydrogels for tissue engineering: a short review. Biomimetics 8 (8), 612. 10.3390/biomimetics8080612 38132551 PMC10742028

[B4] Almine J. F. Bax D. V. Mithieux S. M. Nivison-Smith L. Rnjak J. Waterhouse A. (2010). Elastin-based materials. Chem. Soc. Rev. 39 (9), 3371–3379. 10.1039/b919452p 20449520

[B5] Annabi N. Mithieux S. M. Camci-Unal G. Dokmeci M. R. Weiss A. S. Khademhosseini A. (2013). Elastomeric recombinant protein-based biomaterials. Biochem. Eng. J. 77, 110–118. 10.1016/j.bej.2013.05.006 23935392 PMC3735178

[B6] Antmen E. Vrana N. E. Hasirci V. (2021). The role of biomaterials and scaffolds in immune responses in regenerative medicine: macrophage phenotype modulation by biomaterial properties and scaffold architectures. Biomaterials Sci. 9 (24), 8090–8110. 10.1039/d1bm00840d 34762077

[B7] Antonicelli F. Bellon G. Lorimier S. Hornebeck W. (2009). Role of the elastin receptor complex (S-Gal/Cath-A/Neu-1) in skin repair and regeneration. Wound Repair Regen. 17 (5), 631–638. 10.1111/j.1524-475x.2009.00525.x 19769716

[B8] Aparecido dos Santos-Pinto J. R. Arcuri H. A. Esteves F. G. Palma M. S. Lubec G. (2018). Spider silk proteome provides insight into the structural characterization of *Nephila clavipes* flagelliform spidroin. Sci. Rep. 8, 14674. 10.1038/s41598-018-33068-9 30279551 PMC6168590

[B9] Arabi T. Z. Sabbah B. N. Lerman A. Zhu X.-Y. Lerman L. O. (2023). Xenotransplantation: current challenges and emerging solutions. Cell Transplant. 32, 096368972211487. 10.1177/09636897221148771 PMC9846288 36644844

[B10] Arcidiacono S. Mello C. Kaplan D. Cheley S. Bayley H. (1998). Purification and characterization of recombinant spider silk expressed in *Escherichia coli* . Appl. Microbiol. Biotechnol. 49 (1), 31–38. 10.1007/s002530051133 9487707

[B11] Arif Z. U. Khalid M. Y. Noroozi R. Sadeghianmaryan A. Jalalvand M. Hossain M. (2022). Recent advances in 3D-printed polylactide and polycaprolactone-based biomaterials for tissue engineering applications. Int. J. Biol. Macromol. 218, 930–968. 10.1016/j.ijbiomac.2022.07.140 35896130

[B12] Backlund C. Jalili-Firoozinezhad S. Kim B. Irvine D. J. (2023). Biomaterials-Mediated engineering of the immune system. Annu. Rev. Immunol. 41, 153–179. 10.1146/annurev-immunol-101721-040259 36696570 PMC10375298

[B13] Báez J. Olsen D. Polarek J. W. (2005). Recombinant microbial systems for the production of human collagen and gelatin. Appl. Microbiol. Biotechnol. 69 (3), 245–252. 10.1007/s00253-005-0180-x 16240115

[B14] Bakhshandeh B. Nateghi S. S. Gazani M. M. Dehghani Z. Mohammadzadeh F. (2021). A review on advances in the applications of spider silk in biomedical issues. Int. J. Biol. Macromol. 192, 258–271. 10.1016/j.ijbiomac.2021.09.201 34627845

[B15] Balavigneswaran C. K. Selvaraj S. Vasudha T. K. Iniyan S. Muthuvijayan V. (2023). Tissue engineered skin substitutes: a comprehensive review of basic design, fabrication using 3D printing, recent advances and challenges. Biomater. Adv., 153. 10.1016/j.bioadv.2023.213570 37540939

[B16] Bataille L. Dieryck W. Hocquellet A. Cabanne C. Bathany K. Lecommandoux S. (2015). Expression and purification of short hydrophobic elastin-like polypeptides with maltose-binding protein as a solubility tag. Protein Expr. Purif. 110, 165–171. 10.1016/j.pep.2015.03.013 25819942

[B17] Bataille L. Dieryck W. Hocquellet A. Cabanne C. Bathany K. Lecommandoux S. (2016). Recombinant production and purification of short hydrophobic Elastin-like polypeptides with low transition temperatures. Protein Expr. Purif. 121, 81–87. 10.1016/j.pep.2016.01.010 26802681

[B18] Ben C. Liu X. Shen T. Song Y. Li H. Pan B. (2021). A recombinant human collagen hydrogel for the treatment of partial-thickness burns: a prospective, self-controlled clinical study. Burns 47 (3), 634–642. 10.1016/j.burns.2020.01.006 33402311

[B19] Bessa P. C. Machado R. Nuernberger S. Dopler D. Banerjee A. Cunha A. M. (2010). Thermoresponsive self-assembled elastin-based nanoparticles for delivery of BMPs. J. Control. Release 142 (3), 312–318. 10.1016/j.jconrel.2009.11.003 19913578

[B20] Bitar L. Isella B. Bertella F. Bettker Vasconcelos C. Harings J. Kopp A. (2024). Sustainable *Bombyx mori*'s silk fibroin for biomedical applications as a molecular biotechnology challenge: a review. Int. J. Biol. Macromol. 264 (Pt 1), 130374. 10.1016/j.ijbiomac.2024.130374 38408575

[B21] Brooks A. E. Stricker S. M. Joshi S. B. Kamerzell T. J. Middaugh C. R. Lewis R. V. (2008). Properties of synthetic spider silk fibers based on *Argiope aurantia* MaSp2. Biomacromolecules 9 (6), 1506–1510. 10.1021/bm701124p 18457450

[B22] Buechter D. D. Paolella D. N. Leslie B. S. Brown M. S. Mehos K. A. Gruskin E. A. (2003). Co-Translational incorporation ofTrans-4-hydroxyproline into recombinant proteins in bacteria. J. Biol. Chem. 278 (1), 645–650. 10.1074/jbc.m209364200 12399455

[B23] Bulleid N. J. John D. C. A. Kadler K. E. (2000). Recombinant expression systems for the production of collagen. Biochem. Soc. Trans. 28, 350–353. 10.1042/bst0280350 10961917

[B24] Burnett M. J. B. Burnett A. C. (2020). Therapeutic recombinant protein production in plants: challenges and opportunities. Plants People Planet 2 (2), 121–132. 10.1002/ppp3.10073

[B25] Cabanne C. Pezzini J. Joucla G. Hocquellet A. Barbot C. Garbay B. (2009). Efficient purification of recombinant proteins fused to maltose-binding protein by mixed-mode chromatography. J. Chromatogr. A 1216 (20), 4451–4456. 10.1016/j.chroma.2009.03.048 19329121

[B26] Cankorur-Cetinkaya A. Narraidoo N. Kasavi C. Slater N. K. H. Archer D. B. Oliver S. G. (2018). Process development for the continuous production of heterologous proteins by the industrial yeast, Komagataella phaffii. Biotechnol. Bioeng. 115 (12), 2962–2973. 10.1002/bit.26846 30267565 PMC6283250

[B27] Cao J. Wang P. Liu Y. Zhu C. Fan D. (2020). Double crosslinked HLC-CCS hydrogel tissue engineering scaffold for skin wound healing. Int. J. Biol. Macromol. 155, 625–635. 10.1016/j.ijbiomac.2020.03.236 32240736

[B28] Cao L. Zhang Z. Yuan D. Yu M. Min J. (2024a). Tissue engineering applications of recombinant human collagen: a review of recent progress. Front. Bioeng. Biotechnol. 12, 1358246. 10.3389/fbioe.2024.1358246 38419725 PMC10900516

[B29] Cao L. Zhang Z. Yuan D. Yu M. Min J. (2024b). Tissue engineering applications of recombinant human collagen: a review of recent progress. Front. Bioeng. Biotechnol. 12, 1358246. 10.3389/fbioe.2024.1358246 38419725 PMC10900516

[B30] Cao M. Shen Y. Wang Y. Wang X. Li D. (2019). Self-Assembly of short elastin-like amphiphilic peptides: effects of temperature, molecular hydrophobicity and charge distribution. Molecules 24 (1), 202. 10.3390/molecules24010202 30625991 PMC6337584

[B31] Carvalho V. Domingues L. Gama M. (2008). The inhibitory effect of an RGD-human chitin-binding domain fusion protein on the adhesion of fibroblasts to reacetylated chitosan films. Mol. Biotechnol. 40 (3), 269–279. 10.1007/s12033-008-9089-9 18677572

[B32] Castiglione Morelli M. A. DeBiasi M. DeStradis A. Tamburro A. M. (1993). An aggregating elastin-like pentapeptide. J. Biomol. Struct. Dyn. 11 (1), 181–190. 10.1080/07391102.1993.10508716 8216943

[B33] Celebi B. Cloutier M. Balloni R. Mantovani D. Bandiera A. (2012). Human elastin-based recombinant biopolymers improve mesenchymal stem cell differentiation. Macromol. Biosci. 12 (11), 1546–1554. 10.1002/mabi.201200170 23042756

[B34] Chambre L. Martin-Moldes Z. Parker R. N. Kaplan D. L. (2020). Bioengineered elastin- and silk-biomaterials for drug and gene delivery. Adv. Drug Deliv. Rev. 160, 186–198. 10.1016/j.addr.2020.10.008 33080258 PMC7736173

[B35] Chang M. P. Huang W. Mai D. J. (2021). Monomer-scale design of functional protein polymers using consensus repeat sequences. J. Polym. Sci. 59 (22), 2644–2664. 10.1002/pol.20210506

[B36] Chen W. Li C. Jiang X. (2023b). Advanced biomaterials with intrinsic immunomodulation effects for cancer immunotherapy. Small Methods 7 (5), e2201404. 10.1002/smtd.202201404 36811240

[B37] Chen Y. Wu Y. Xiong F. Yu W. Wang T. Xiong J. (2023a). Construction of a collagen-like protein based on elastin-like polypeptide fusion and evaluation of its performance in promoting wound healing. Molecules 28 (19), 6773. 10.3390/molecules28196773 37836616 PMC10574607

[B38] Chen Z. Fan D. Shang L. (2021). Exploring the potential of the recombinant human collagens for biomedical and clinical applications: a short review. Biomed. Mater. 16 (1), 012001. 10.1088/1748-605x/aba6fa 32679570

[B39] Cheng Y. Li Y. Huang S. Yu F. Bei Y. Zhang Y. (2020). Hybrid freeze-dried dressings composed of epidermal growth factor and recombinant human-like collagen enhance cutaneous wound healing in rats. Front. Bioeng. Biotechnol. 8, 742. 10.3389/fbioe.2020.00742 32760705 PMC7375021

[B40] Choi S. M. Chaudhry P. Zo S. M. Han S. S. (2018). Delivery of bioactive agents from recombinant polymers. Prog. Polym. Sci. 32 (8-9), 1008–1030. 10.1016/j.progpolymsci.2007.05.015

[B41] Chouhan D. Lohe T.-u. Thatikonda N. Naidu V. G. M. Hedhammar M. Mandal B. B. (2019). Silkworm silk scaffolds functionalized with recombinant spider silk containing a fibronectin motif promotes healing of full-thickness burn wounds. Acs Biomaterials Sci. and Eng. 5 (9), 4634–4645. 10.1021/acsbiomaterials.9b00887 33448836

[B42] Chouhan D. Mandal B. B. (2020). Silk biomaterials in wound healing and skin regeneration therapeutics: from bench to bedside. Acta Biomater. 103, 24–51. 10.1016/j.actbio.2019.11.050 31805409

[B43] Chouhan D. Thatikonda N. Nileback L. Widhe M. Hedhammar M. Mandal B. B. (2018). Recombinant spider silk functionalized silkworm silk matrices as potential bioactive wound dressings and skin grafts. Acs Appl. Mater. and Interfaces 10 (28), 23560–23572. 10.1021/acsami.8b05853 29940099

[B44] Ciofani G. Genchi G. G. Guardia P. Mazzolai B. Mattoli V. Bandiera A. (2014). Recombinant human elastin-like magnetic microparticles for drug delivery and targeting. Macromol. Biosci. 14 (5), 632–642. 10.1002/mabi.201300361 24318291

[B45] Collin M. A. Clarke T. H. III Ayoub N. A. Hayashi C. Y. (2018). Genomic perspectives of spider silk genes through target capture sequencing: conservation of stabilization mechanisms and homology-based structural models of spidroin terminal regions. Int. J. Biol. Macromol. 113, 829–840. 10.1016/j.ijbiomac.2018.02.032 29454054

[B46] Daamen W. F. Nillesen S. T. M. Wismans R. G. Reinhardt D. P. Hafmans T. Veerkamp J. H. (2008). A biomaterial composed of collagen and solubilized elastin enhances angiogenesis and elastic fiber formation without calcification. Tissue Eng. Part A 14 (3), 349–360. 10.1089/tea.2007.0076 18333787

[B47] da Costa A. Machado R. Ribeiro A. Collins T. Thiagarajan V. Neves-Petersen M. T. (2015). Development of elastin-like recombinamer films with antimicrobial activity. Biomacromolecules 16 (2), 625–635. 10.1021/bm5016706 25580615

[B48] Dandu R. Ghandehari H. (2007). Delivery of bioactive agents from recombinant polymers. Prog. Polym. Sci. 32 (8-9), 1008–1030. 10.1016/j.progpolymsci.2007.05.015

[B49] Davison-Kotler E. Marshall W. S. Garcia-Gareta E. (2019). Sources of collagen for biomaterials in skin wound healing. Bioengineering-Basel 6 (3), 56. 10.3390/bioengineering6030056 31261996 PMC6783949

[B50] De Giorgio G. Matera B. Vurro D. Manfredi E. Galstyan V. Tarabella G. (2024). Silk fibroin materials: biomedical applications and perspectives. Bioengineering-Basel 11 (2), 167. 10.3390/bioengineering11020167 38391652 PMC10886036

[B51] Deng A. Yang Y. Du S. Yang S. (2018). Electrospinning of *in situ* crosslinked recombinant human collagen peptide/chitosan nanofibers for wound healing. Biomaterials Sci. 6 (8), 2197–2208. 10.1039/c8bm00492g 30003209

[B52] de Souza A. Martignago C. C. S. Santo G. d. E. Sousa K. d. S. J. Cruz M. A. Amaral G. O. (2023). 3D printed wound constructs for skin tissue engineering: a systematic review in experimental animal models. J. Biomed. Mater. Res. Part B-Applied Biomaterials 111 (7), 1419–1433. 10.1002/jbm.b.35237 36840674

[B53] DiMarco R. L. Heilshorn S. C. (2012). Multifunctional materials through modular protein engineering. Adv. Mater. 24 (29), 3923–3940. 10.1002/adma.201200051 22730248

[B54] Dinjaski N. Kaplan D. L. (2016). Recombinant protein blends: silk beyond natural design. Curr. Opin. Biotechnol. 39, 1–7. 10.1016/j.copbio.2015.11.002 26686863

[B55] Dinjaski N. Plowright R. Zhou S. Belton D. J. Perry C. C. Kaplan D. L. (2017). Osteoinductive recombinant silk fusion proteins for bone regeneration. Acta Biomater. 49, 127–139. 10.1016/j.actbio.2016.12.002 27940162 PMC5253115

[B56] Doloff J. C. Veiseh O. de Mezerville R. Sforza M. Perry T. A. Haupt J. (2021). The surface topography of silicone breast implants mediates the foreign body response in mice, rabbits and humans. Nat. Biomed. Eng. 5 (10), 1115–1130. 10.1038/s41551-021-00739-4 34155355

[B57] Dong J. Wang W. Zhou W. Zhang S. Li M. Li N. (2022). Immunomodulatory biomaterials for implant-associated infections: from conventional to advanced therapeutic strategies. Biomaterials Res. 26 (1), 72. 10.1186/s40824-022-00326-x PMC9721013 36471454

[B58] Dong Z. Liu Q. Han X. Zhang X. Wang X. Hu C. (2023). Electrospun nanofibrous membranes of recombinant human collagen type III promote cutaneous wound healing. J. Mater. Chem. B 11 (27), 6346–6360. 10.1039/d3tb00438d 37309213

[B59] Fahnestock S. R. Bedzyk L. A. (1997). Production of synthetic spider dragline silk protein in Pichia pastoris. Appl. Microbiol. Biotechnol. 47 (1), 33–39. 10.1007/s002530050884 9035408

[B60] Fahnestock S. R. Irwin S. L. (1997). Synthetic spider dragline silk proteins and their production in *Escherichia coli* . Appl. Microbiol. Biotechnol. 47 (1), 23–32. 10.1007/s002530050883 9035407

[B61] Fan C. Feng L. Fan J. Guo D. Liu X. (2012). Recent advances on the expression systems for recombinant protein production. Biotechnology 22 (2), 76–80.

[B62] Fang J. Ma Z. Liu D. Wang Z. Cheng S. Zheng S. (2023). Co-expression of recombinant human collagen α1(III) chain with viral prolyl 4-hydroxylase in Pichia pastoris GS115. Protein Expr. Purif. 201, 106184. 10.1016/j.pep.2022.106184 36191842

[B63] Faravelli S. Campioni M. Palamini M. Canciani A. Chiapparino A. Forneris F. (2021). Optimized recombinant production of secreted proteins using human embryonic kidney (HEK293) cells grown in suspension. Bio-Protocol 11 (8), e3998. 10.21769/bioprotoc.3998 34124299 PMC8160536

[B64] Feng Z. Wang S. Huang W. Bai W. (2024). A potential bilayer skin substitute based on electrospun silk-elastin-like protein nanofiber membrane covered with bacterial cellulose. Colloids Surfaces B-Biointerfaces 234, 113677. 10.1016/j.colsurfb.2023.113677 38043505

[B65] Fertala A. (2020). Three decades of research on recombinant collagens: reinventing the wheel or developing new biomedical products? Bioengineering-Basel 7 (4), 155. 10.3390/bioengineering7040155 33276472 PMC7712652

[B66] Fertala A. Sieron A. L. Ganguly A. Li S. W. Alakokko L. Anumula K. R. (1994). Synthesis of recombinant human procollagen II in a stably transfected tumour cell line (HT1080). Biochem. J. 298, 31–37. 10.1042/bj2980031 8129728 PMC1137979

[B67] Foo C. W. P. Kaplan D. L. (2002). Genetic engineering of fibrous proteins: spider dragline silk and collagen. Adv. Drug Deliv. Rev. 54 (8), 1131–1143. 10.1016/s0169-409x(02)00061-3 12384311

[B68] Garcia C. G. Patkar S. S. Wang B. Abouomar R. Kiick K. L. (2023). Recombinant protein-based injectable materials for biomedical applications. Adv. Drug Deliv. Rev. 193, 114673. 10.1016/j.addr.2022.114673 36574920

[B69] Gardeazabal L. Izeta A. (2024). Elastin and collagen fibres in cutaneous wound healing. Exp. Dermatol. 33 (3), e15052. 10.1111/exd.15052 38483134

[B70] Gecchele E. Merlin M. Brozzetti A. Falorni A. Pezzotti M. Avesani L. (2015). A comparative analysis of recombinant protein expression in different biofactories: bacteria, insect cells and plant systems. Jove-Journal Vis. Exp. 97. 10.3791/52459-v PMC4401374 25867956

[B71] Geddis A. E. Prockop D. J. (1993). Expression of human COL1A1 gene in stably transfected HT1080 cells: the production of a thermostable homotrimer of type I collagen in a recombinant system. Matrix 13 (5), 399–405. 10.1016/s0934-8832(11)80045-4 8246835

[B72] Geisler C. Jarvis D. L. (2018). Adventitious viruses in insect cell lines used for recombinant protein expression. Protein Expr. Purif. 144, 25–32. 10.1016/j.pep.2017.11.002 29133148 PMC5826799

[B73] Gelse K. Pöschl E. Aigner T. (2003). Collagens -: structure, function, and biosynthesis. Adv. Drug Deliv. Rev. 55 (12), 1531–1546. 10.1016/j.addr.2003.08.002 14623400

[B74] Gholipourmalekabadi M. Sapru S. Samadikuchaksaraei A. Reis R. L. Kaplan D. L. Kundu S. C. (2020). Silk fibroin for skin injury repair: where do things stand? Adv. Drug Deliv. Rev. 153, 28–53. 10.1016/j.addr.2019.09.003 31678360

[B75] Gomes S. Numata K. Leonor I. B. Mano J. F. Reis R. L. Kapan D. L. (2011). AFM study of morphology and mechanical properties of a chimeric spider silk and bone sialoprotein protein for bone regeneration. Biomacromolecules 12 (5), 1675–1685. 10.1021/bm2000605 21370930 PMC3090475

[B76] Grip S. Rising A. Nimmervoll H. Storckenfeldt E. McQueen-Mason S. J. Pouchkina-Stantcheva N. (2006). Transient expression of a major ampullate spidroin 1 gene fragment from <i>Euprosthenops</i> sp. in mammalian cells. Cancer Genomics and Proteomics 3 (2), 83–87. 31394685

[B77] Guo X. Ma Y. Wang H. Yin H. Shi X. Chen Y. (2024). Status and developmental trends in recombinant collagen preparation technology. Regen. Biomater. 11, rbad106. 10.1093/rb/rbad106 38173768 PMC10761200

[B78] Guo Y. Bian Z. Xu Q. Wen X. Kang J. Lin S. (2021). Novel tissue-engineered skin equivalent from recombinant human collagen hydrogel and fibroblasts facilitated full-thickness skin defect repair in a mouse model. Mater. Sci. Eng. C-Materials Biol. Appl. 130, 112469. 10.1016/j.msec.2021.112469 34702544

[B79] Guo Y. Xu B. Wang Y. Li Y. Si H. Zheng X. (2019). Dramatic promotion of wound healing using a recombinant human-like collagen and bFGF cross-linked hydrogel by transglutaminase. J. Biomaterials Science-Polymer Ed. 30 (17), 1591–1603. 10.1080/09205063.2019.1652416 31411556

[B80] Hardy J. G. Roemer L. M. Scheibel T. R. (2008). Polymeric materials based on silk proteins. Polymer 49 (20), 4309–4327. 10.1016/j.polymer.2008.08.006

[B81] Hayashi M. Tomita M. Yoshizato K. (2001). Production of EGF-collagen chimeric protein which shows the mitogenic activity. Biochimica Biophysica Acta-General Subj. 1528 (2-3), 187–195. 10.1016/s0304-4165(01)00187-8 11687306

[B82] He J. Ma X. Zhang F. Li L. Deng J. Xue W. (2015). New strategy for expression of recombinant hydroxylated human collagen 1(III) chains in *Pichia pastoris* GS115. Biotechnol. Appl. Biochem. 62 (3), 293–299. 10.1002/bab.1264 24953863

[B83] He Y.-X. Zhang N.-N. Li W.-F. Jia N. Chen B.-Y. Zhou K. (2012). N-terminal domain of *Bombyx mori* fibroin mediates the assembly of silk in response to pH decrease. J. Mol. Biol. 418 (3-4), 197–207. 10.1016/j.jmb.2012.02.040 22387468

[B84] Heidebrecht A. Eisoldt L. Diehl J. Schmidt A. Geffers M. Lang G. (2015). Biomimetic fibers made of recombinant spidroins with the same toughness as natural spider silk. Adv. Mater. 27 (13), 2189–2194. 10.1002/adma.201404234 25689835

[B85] Heidebrecht A. Scheibel T. (2013). Purification and characterization of recombinant spider silk expressed in *Escherichia coli* . Appl. Microbiol. Biotechnol. 49 (1), 31–38. 10.1007/s002530051133 9487707

[B86] Hinek A. Bodnaruk T. D. Bunda S. Wang Y. Liu K. (2008). Neuraminidase-1, a subunit of the cell surface elastin receptor, desialylates and functionally inactivates adjacent receptors interacting with the mitogenic growth factors PDGF-BB and IGF-2. Am. J. Pathol. 173 (4), 1042–1056. 10.2353/ajpath.2008.071081 18772331 PMC2543072

[B87] Hu X. Vasanthavada K. Kohler K. McNary S. Moore A. M. F. Vierra C. A. (2006). Molecular mechanisms of spider silk. Cell. Mol. Life Sci. 63 (17), 1986–1999. 10.1007/s00018-006-6090-y 16819558 PMC11136436

[B88] Huang J. Lei X. Huang Z. Rong Z. Li H. Xie Y. (2022). Bioprinted gelatin-recombinant type III collagen hydrogel promotes wound healing. Int. J. Bioprinting 8 (2), 517–524. 10.18063/ijb.v8i2.517 PMC9159484 35669327

[B89] Huemmerich D. Scheibel T. Vollrath F. Cohen S. Gat U. Ittah S. (2004). Novel assembly properties of recombinant spider dragline silk proteins. Curr. Biol. 14 (22), 2070–2074. 10.1016/j.cub.2004.11.005 15556872

[B90] Humenik M. Magdeburg M. Scheibel T. (2014). Influence of repeat numbers on self-assembly rates of repetitive recombinant spider silk proteins. J. Struct. Biol. 186 (3), 431–437. 10.1016/j.jsb.2014.03.010 24657229

[B91] Humenik M. Mohrand M. Scheibel T. (2018). Self-Assembly of spider silk-fusion proteins comprising enzymatic and fluorescence activity. Bioconjugate Chem. 29 (4), 898–904. 10.1021/acs.bioconjchem.7b00759 29338201

[B92] Incir I. Kaplan O. (2024). *Escherichia coli* as a versatile cell factory: advances and challenges in recombinant protein production. Protein Expr. Purif. 219, 106463. 10.1016/j.pep.2024.106463 38479588

[B93] Isaacson K. J. Jensen M. M. Watanabe A. H. Green B. E. Correa M. A. Cappello J. (2018). Self-Assembly of thermoresponsive recombinant silk-elastinlike nanogels. Macromol. Biosci. 18 (1). 10.1002/mabi.201700192 PMC5806626 28869362

[B94] Jenkins I. C. Milligan J. J. Chilkoti A. (2021). Genetically encoded elastin-like polypeptides for drug delivery. Adv. Healthc. Mater. 10 (13), e2100209. 10.1002/adhm.202100209 34080796

[B95] John D. C. A. Watson R. Kind A. J. Scott A. R. Kadler K. E. Bulleid N. J. (1999). Expression of an engineered form of recombinant procollagen in mouse milk. Nat. Biotechnol. 17 (4), 385–389. 10.1038/7945 10207889

[B96] Kahan V. Andersen M. L. Tomimori J. Tufik S. (2009). Stress, immunity and skin collagen integrity: evidence from animal models and clinical conditions. Brain Behav. Immun. 23 (8), 1089–1095. 10.1016/j.bbi.2009.06.002 19523511

[B97] Kang D. Wang W. Li Y. Ma Y. Huang Y. Wang J. (2023). Biological macromolecule hydrogel based on recombinant type I collagen/chitosan scaffold to accelerate full-thickness healing of skin wounds. Polymers 15 (19), 3919. 10.3390/polym15193919 37835967 PMC10575414

[B98] Kang H. J. Kumar S. D'Elia A. Dash B. Nanda V. Hsia H. C. (2021). Self-assembled elastin-like polypeptide fusion protein coacervates as competitive inhibitors of advanced glycation end-products enhance diabetic wound healing. J. Control. Release 333, 176–187. 10.1016/j.jconrel.2021.03.032 33781808 PMC10927318

[B99] Karahisar Turan S. Kilic Suloglu A. Ide S. Turkes T. Barlas N. (2024). *In vitro* and *in vivo* investigation of Argiope bruennichi spider silk-based novel biomaterial for medical use. Biopolymers 115, e23572. 10.1002/bip.23572 38491802

[B100] Karbalaei M. Rezaee S. A. Farsiani H. (2020a). *Pichia pastoris*: a highly successful expression system for optimal synthesis of heterologous proteins. J. Cell. Physiology 235 (9), 5867–5881. 10.1002/jcp.29583 PMC7228273 32057111

[B101] Karbalaei M. Rezaee S. A. Farsiani H. (2020b). Pichia pastoris: a highly successful expression system for optimal synthesis of heterologous proteins. J. Cell Physiol. 235 (9), 5867–5881. 10.1002/jcp.29583 32057111 PMC7228273

[B102] Karle I. L. Urry D. W. (2005). Crystal structure of cyclic (APGVGV)2, an analog of elastin, and a suggested mechanism for elongation/contraction of the molecule. Biopolymers 77 (4), 198–204. 10.1002/bip.20214 15666330

[B103] Kaur J. Kumar A. Kaur J. (2018a). Strategies for optimization of heterologous protein expression in *E-coli:* roadblocks and reinforcements. Int. J. Biol. Macromol. 106, 803–822. 10.1016/j.ijbiomac.2017.08.080 28830778

[B104] Kaur J. Kumar A. Kaur J. (2018b). Strategies for optimization of heterologous protein expression in *E. coli*: roadblocks and reinforcements. Int. J. Biol. Macromol. 106, 803–822. 10.1016/j.ijbiomac.2017.08.080 28830778

[B105] Khalili S. Khorasani S. N. Razavi S. M. Hashemibeni B. Tamayol A. (2019). Nanofibrous scaffolds with biomimetic composition for skin regeneration. Appl. Biochem. Biotechnol. 187 (4), 1193–1203. 10.1007/s12010-018-2871-7 30187343

[B106] Kim B. S. Kwon Y. W. Kong J.-S. Park G. T. Gao G. Han W. (2018). 3D cell printing of *in vitro* stabilized skin model and *in vivo* pre-vascularized skin patch using tissue-specific extracellular matrix bioink: a step towards advanced skin tissue engineering. Biomaterials 168, 38–53. 10.1016/j.biomaterials.2018.03.040 29614431

[B107] Kim H.-W. Kang W. Jeon E. Jang J.-H. (2010). Construction and expression of a recombinant fibronectin_III_10 protein for integrin-mediated cell adhesion. Biotechnol. Lett. 32 (1), 29–33. 10.1007/s10529-009-0111-5 19707723

[B108] Kinikoglu B. Carlos Rodriguez-Cabello J. Damour O. Hasirci V. (2011). A smart bilayer scaffold of elastin-like recombinamer and collagen for soft tissue engineering. J. Mater. Science-Materials Med. 22 (6), 1541–1554. 10.1007/s10856-011-4315-6 21505829

[B109] Kljenak A. Trcin M. T. Bujic M. Dolenec T. Jevak M. Mrsic G. (2016). FIBRIN GEL AS A SCAFFOLD FOR SKIN SUBSTITUTE - PRODUCTION AND CLINICAL EXPERIENCE. Acta Clin. Croat. 55 (2), 279–289. 10.20471/acc.2016.55.02.15 28394544

[B110] Knight D. P. Vollrath F. (2001). Changes in element composition along the spinning duct in a Nephila spider. Naturwissenschaften 88 (4), 179–182. 10.1007/s001140100220 11480706

[B111] Kourtzelis I. Rafail S. DeAngelis R. A. Foukas P. G. Ricklin D. Lambris J. D. (2013). Inhibition of biomaterial-induced complement activation attenuates the inflammatory host response to implantation. Faseb J. 27 (7), 2768–2776. 10.1096/fj.12-225888 23558338 PMC3688753

[B112] Kramer R. Z. Bella J. Mayville P. Brodsky B. Berman H. M. (1999). Sequence dependent conformational variations of collagen triple-helical structure. Nat. Struct. Biol. 6 (5), 454–457. 10.1038/8259 10331873

[B113] Kundu B. Rajkhowa R. Kundu S. C. Wang X. (2013). Silk fibroin biomaterials for tissue regenerations. Adv. Drug Deliv. Rev. 65 (4), 457–470. 10.1016/j.addr.2012.09.043 23137786

[B114] Kuwana Y. Sezutsu H. Nakajima K.-i. Tamada Y. Kojima K. (2014). High-toughness silk produced by a transgenic silkworm expressing spider (*Araneus ventricosus*) dragline silk protein. Plos One 9 (8), e105325. 10.1371/journal.pone.0105325 25162624 PMC4146547

[B115] Lamberg A. Helaakoski T. Myllyharju J. Peltonen S. Notbohm H. Pihlajaniemi T. (1996). Characterization of human type III collagen expressed in a baculovirus system. J. Biol. Chem. 271 (20), 11988–11995. 10.1074/jbc.271.20.11988 8662631

[B116] La Monica F. Campora S. Ghersi G. (2024). Collagen-based scaffolds for chronic skin wound treatment. Gels 10 (2), 137. 10.3390/gels10020137 38391467 PMC10888252

[B117] Lawrence B. A. Vierra C. A. Mooref A. M. F. (2004). Molecular and mechanical properties of major ampullate silk of the black widow spider, *Latrodectus hesperus* . Biomacromolecules 5 (3), 689–695. 10.1021/bm0342640 15132648

[B118] Lebre F. Hearnden C. H. Lavelle E. C. (2016). Modulation of immune responses by particulate materials. Adv. Mater. 28 (27), 5525–5541. 10.1002/adma.201505395 27167228

[B119] Ledford B. Barron C. Van Dyke M. He J. Q. (2022). Keratose hydrogel for tissue regeneration and drug delivery. Seminars Cell and Dev. Biol. 128, 145–153. 10.1016/j.semcdb.2021.06.017 34219034

[B120] Le Fer G. Wirotius A.-L. Brulet A. Garanger E. Lecommandoux S. (2019). Self-Assembly of stimuli-responsive biohybrid synthetic-b-recombinant block copolypeptides. Biomacromolecules 20 (1), 254–272. 10.1021/acs.biomac.8b01390 30458105

[B121] Lewis R. V. Xia L. Zhang X. Jones J. A. (2021) Transgenic silkworms expressing spider silk, 11089767. US: Patent class: A01K670335.

[B122] Li J. Huang W. He H. Shi S. Sun X. Xiao J. (2023a). Biocompatible and bioactive hydrogels of recombinant fusion elastin with low transition temperature for improved healing of UV-irradiated skin. J. Mater. Chem. B 11 (29), 6975–6982. 10.1039/d3tb00564j 37401183

[B123] Li J. N. Huang W. J. He H. X. Shi S. N. Sun X. X. Xiao J. X. (2023b). Biocompatible and bioactive hydrogels of recombinant fusion elastin with low transition temperature for improved healing of UV-irradiated skin. J. Mater. Chem. B 11 (29), 6975–6982. 10.1039/d3tb00564j 37401183

[B124] Li L. Tong Z. Jia X. Kiick K. L. (2013b). Resilin-like polypeptide hydrogels engineered for versatile biological function. Soft Matter 9 (3), 665–673. 10.1039/c2sm26812d 23505396 PMC3595062

[B125] Li R. Liu K. Huang X. Li D. Ding J. Liu B. (2022). Bioactive materials promote wound healing through modulation of cell behaviors. Adv. Sci. 9 (10), e2105152. 10.1002/advs.202105152 PMC8981489 35138042

[B126] Li Z.-H. Ji S.-C. Wang Y.-Z. Shen X.-C. Liang H. (2013a). Silk fibroin-based scaffolds for tissue engineering. Front. Mater. Sci. 7 (3), 237–247. 10.1007/s11706-013-0214-8

[B127] Lian J. Ju G. Cai X. Cai Y. Li C. Ma S. (2021). Nanofibrous membrane dressings loaded with sodium hydrogen sulfide/endothelial progenitor cells promote wound healing. Front. Bioeng. Biotechnol. 9, 657549. 10.3389/fbioe.2021.657549 34422776 PMC8372243

[B128] Liu D. Wang F. Li W. (2011). Cloning and expression of spider dragline silk protein gene in *Escherichia coli* and eukaryotic cells. Genomics Appl. Biol. 30 (1), 16–20. 10.5376/ME.2011.02.0001

[B129] Liu H. Dong J. Du R. Gao Y. Zhao P. (2023a). Collagen study advances for photoaging skin. Photodermatol. Photoimmunol. and Photomed. 40, e12931. 10.1111/phpp.12931 38009842

[B130] Liu S. Li Y. Wang M. Ma Y. Wang J. (2023b). Efficient coexpression of recombinant human fusion collagen with prolyl 4‐hydroxylase from *Bacillus anthracis* in *Escherichia coli* . Biotechnol. Appl. Biochem. 70 (2), 761–772. 10.1002/bab.2396 35959739

[B131] Liu T. Qiu C. Lu H. Li H. Zhu S. Ma L. (2023c). A novel recombinant human collagen hydrogel as minced split-thickness skin graft overlay to promote full-thickness skin defect reconstruction. Burns 49 (1), 169–181. 10.1016/j.burns.2022.02.015 35361497

[B132] Lojewska E. Kowalczyk T. Olejniczak S. Sakowicz T. (2016). Extraction and purification methods in downstream processing of plant-based recombinant proteins. Protein Expr. Purif. 120, 110–117. 10.1016/j.pep.2015.12.018 26742898

[B133] Long L.-y. Liu W. Li L. Hu C. He S. Lu L. (2022). Dissolving microneedle-encapsulated drug-loaded nanoparticles and recombinant humanized collagen type III for the treatment of chronic wound via anti-inflammation and enhanced cell proliferation and angiogenesis. Nanoscale 14 (4), 1285–1295. 10.1039/d1nr07708b 35006234

[B134] Lovell C. R. Smolenski K. A. Duance V. C. Light N. D. Young S. Dyson M. (1987). Type I and III collagen content and fibre distribution in normal human skin during ageing. Br. J. Dermatology 117 (4), 419–428. 10.1111/j.1365-2133.1987.tb04921.x 3676091

[B135] Low P. S. J. Tjin M. S. Fong E. (2015). Design and construction of artificial extracellular matrix (aECM) proteins from *Escherichia coli* for skin tissue engineering. Jove-Journal Vis. Exp. 100, e52845. 10.3791/52845 PMC4545042 26132812

[B136] Ma L. Liang X. Yu S. Zhou J. (2022). Expression, characterization, and application potentiality evaluation of recombinant human-like collagen in Pichia pastoris. Bioresour. Bioprocess. 9 (1), 119. 10.1186/s40643-022-00606-3 38647896 PMC10992492

[B137] Macek B. Forchhammer K. Hardouin J. Weber-Ban E. Grangeasse C. Mijakovic I. (2019). Protein post-translational modifications in bacteria. Nat. Rev. Microbiol. 17 (11), 651–664. 10.1038/s41579-019-0243-0 31485032

[B138] Machado R. da Costa A. Sencadas V. Garcia-Arevalo C. Costa C. M. Padrao J. (2013). Electrospun silk-elastin-like fibre mats for tissue engineering applications. Biomed. Mater. 8 (6), 065009. 10.1088/1748-6041/8/6/065009 24287397

[B139] Mandal A. Pal D. Agrahari V. Hoang My T. Joseph M. Mitra A. K. (2018). Ocular delivery of proteins and peptides: challenges and novel formulation approaches. Adv. Drug Deliv. Rev. 126, 67–95. 10.1016/j.addr.2018.01.008 29339145 PMC5995646

[B140] Manoharan C. Thomas D. S. Yashwant R. S. Mudagal M. P. Janadri S. Roy G. (2022). Bioengineered and functionalized silk proteins accelerate wound healing in rat and human dermal fibroblasts. Integr. Biol. 14 (7), 151–161. 10.1093/intbio/zyac014 36314040

[B141] Mashiko T. Takada H. Wu S.-H. Kanayama K. Feng J. Tashiro K. (2018). Therapeutic effects of a recombinant human collagen peptide bioscaffold with human adipose-derived stem cells on impaired wound healing after radiotherapy. J. Tissue Eng. Regen. Med. 12 (5), 1186–1194. 10.1002/term.2647 29377539

[B142] Mayer A. F. Hellmuth K. Schlieker H. Lopez-Ulibarri R. Oertel S. Dahlems U. (1999). An expression system matures: a highly efficient and cost-effective process for phytase production by recombinant strains ofHansenula polymorpha. Biotechnol. Bioeng. 63 (3), 373–381. 10.1002/(sici)1097-0290(19990505)63:3<373::aid-bit14>3.0.co;2-t 10099617

[B143] McPherson D. T. Xu J. Urry D. W. (1996). Product purification by reversible phase transition following *Escherichia coli* expression of genes encoding up to 251 repeats of the elastomeric pentapeptide GVGVP. Protein Expr. Purif. 7 (1), 51–57. 10.1006/prep.1996.0008 9172783

[B144] Meek M. F. Jansen K. (2009). Two years after <i>*in vivo* implantation of poly(DL-lactide-ε-caprolactone) nerve guides: has the material finally resorbed? J. Biomed. Mater. Res. Part A 89A (3), 734–738. 10.1002/jbm.a.32024 18464254

[B145] Meyer D. E. Chilkoti A. (1999a). Purification of recombinant proteins by fusion with thermally-responsive polypeptides. Nat. Biotechnol. 17 (11), 1112–1115. 10.1038/15100 10545920

[B146] Meyer D. E. Chilkoti A. (1999b). Purification of recombinant proteins by fusion with thermally-responsive polypeptides. Nat. Biotechnol. 17 (11), 1112–1115. 10.1038/15100 10545920

[B147] Meyer D. E. Chilkoti A. (2004). Quantification of the effects of chain length and concentration on the thermal behavior of elastin-like polypeptides. Biomacromolecules 5 (3), 846–851. 10.1021/bm034215n 15132671

[B148] Moire L. Rezzonico E. Poirier Y. (2003). Synthesis of novel biomaterials in plants. J. Plant Physiology 160 (7), 831–839. 10.1078/0176-1617-01030 12940550

[B149] Monfort D. A. Koria P. (2017). Recombinant elastin-based nanoparticles for targeted gene therapy. Gene Ther. 24 (10), 610–620. 10.1038/gt.2017.54 28681841 PMC5658264

[B150] Mortimer J. C. (2019). Plant synthetic biology could drive a revolution in biofuels and medicine. Exp. Biol. Med. 244 (4), 323–331. 10.1177/1535370218793890 PMC6435885 30249124

[B151] Muiznieks L. D. Keeley F. W. (2016). Phase separation and mechanical properties of an elastomeric biomaterial from spider wrapping silk and elastin block copolymers. Biopolymers 105 (10), 693–703. 10.1002/bip.22888 27272259

[B152] Myllyharju J. Lamberg A. Notbohm H. Fietzek P. P. Pihlajaniemi T. Kivirikko K. I. (1997). Expression of wild-type and modified proα chains of human type I procollagen in insect cells leads to the formation of stable [α1(I)]2α2(I) collagen heterotrimers and [α1(I)]3 homotrimers but not [α2(I)]3 homotrimers. J. Biol. Chem. 272 (35), 21824–21830. 10.1074/jbc.272.35.21824 9268313

[B153] Myllyharju J. Nokelainen M. Vuorela A. Kivirikko K. I. (2000). Expression of recombinant human type I‒III collagens in the yeast Pichia pastoris. Biochem. Soc. Trans. 28, 353–357. 10.1042/0300-5127:0280353 10961918

[B154] Nagano A. Tanioka Y. Sakurai N. Sezutsu H. Kuboyama N. Kiba H. (2011). Regeneration of the femoral epicondyle on calcium-binding silk scaffolds developed using transgenic silk fibroin produced by transgenic silkworm. Acta Biomater. 7 (3), 1192–1201. 10.1016/j.actbio.2010.10.032 21055485

[B155] Norahan M. H. Pedroza-Gonz S. C. Sanchez-Salazar M. G. Alvarez M. M. Santiago G. T. d. (2023). Structural and biological engineering of 3D hydrogels for wound healing. Bioact. Mater. 24, 197–235. 10.1016/j.bioactmat.2022.11.019 36606250 PMC9803907

[B156] Olsen A. S. Geddis A. E. Prockop D. J. (1991). High levels of expression of a minigene version of the human pro alpha 1 (I) collagen gene in stably transfected mouse fibroblasts. Effects of deleting putative regulatory sequences in the first intron. J. Biol. Chem. 266 (2), 1117–1121. 10.1016/s0021-9258(17)35290-0 1702430

[B157] Park C. H. Woo K. M. (2018). A comparative study of materials assembled from recombinant K31 and K81 and extracted human hair keratins. Biomed. Mater. 15 (6), 065006. 10.1088/1748-605x/ab98e8 32485704

[B158] Parker R. N. Trent A. Roth Stefaniak K. L. Van Dyke M. E. Grove T. Z. (2020). A comparative study of materials assembled from recombinant K31 and K81 and extracted human hair keratins. Biomed. Mater. 15 (6), 065006. 10.1088/1748-605x/ab98e8 32485704

[B159] Patel J. Willis J. Aluri A. Awad S. Smith M. Banker Z. (2021). Three-dimensionally printed skin substitute using human dermal fibroblasts and human epidermal keratinocytes. Ann. Plastic Surg. 86 (6S), S628–S631. 10.1097/sap.0000000000002886 34100824

[B160] Patkar S. S. Wang B. Mosquera A. M. Kiick K. L. (2024). Genetically fusing order-promoting and thermoresponsive building blocks to design hybrid biomaterials. Chem. (Weinheim der Bergstrasse, Ger.) 30, e202400582. e202400582-e202400582. 10.1002/chem.202400582 PMC11661552 38501912

[B161] Peng Z. Xie C. Jin S. Hu J. Yao X. Ye J. (2023). Biomaterial based implants caused remote liver fatty deposition through activated blood-derived macrophages. Biomaterials 301, 122234. 10.1016/j.biomaterials.2023.122234 37421671

[B162] Pennisi E. Service R. F. (2017). A spinner's secrets. Sci. (New York, N.Y.) 358 (6361), 292. 10.1126/science.358.6361.292 29051359

[B163] Petitdemange R. Garanger E. Bataille L. Bathany K. Garbay B. Deming T. J. (2017). Tuning thermoresponsive properties of cationic elastin-like polypeptides by varying counterions and side-chains. Bioconjugate Chem. 28 (5), 1403–1412. 10.1021/acs.bioconjchem.7b00082 28381088

[B164] Plowright R. Dinjaski N. Zhou S. Belton D. J. Kaplan D. L. Perry C. C. (2016). Influence of silk–silica fusion protein design on silica condensation *in vitro* and cellular calcification. Rsc Adv. 6 (26), 21776–21788. 10.1039/c6ra03706b 26989487 PMC4792305

[B165] Poza P. Pérez-Rigueiro J. Elices M. Llorca J. (2002). Fractographic analysis of silkworm and spider silk. Eng. Fract. Mech. 69 (9), 1035–1048. 10.1016/s0013-7944(01)00120-5

[B166] Prockop D. J. Fertala A. (1998). Inhibition of the self-assembly of collagen I into fibrils with synthetic peptides. J. Biol. Chem. 273 (25), 15598–15604. 10.1074/jbc.273.25.15598 9624151

[B167] Qian Z.-G. Pan F. Xia X.-X. (2020). Synthetic biology for protein-based materials. Curr. Opin. Biotechnol. 65, 197–204. 10.1016/j.copbio.2020.04.004 32492515

[B168] Qin J. Chen F. Wu P. Sun G. (2022). Recent advances in bioengineered scaffolds for cutaneous wound healing. Front. Bioeng. Biotechnol. 10, 841583. 10.3389/fbioe.2022.841583 35299645 PMC8921732

[B169] Rezaei M. Davani F. Alishahi M. Masjedi F. (2022). Updates in immunocompatibility of biomaterials: applications for regenerative medicine. Expert Rev. Med. Devices 19 (4), 353–367. 10.1080/17434440.2022.2075730 35531761

[B170] Ricard-Blum S. Ruggiero F. van der Rest M. (2005). “The collagen superfarmily,”. Collagen: primer in structure, processing and assembly. Editors Brinckmann J. Notbohm H. Muller P. K., 247, 35–84.

[B171] Rice J. W. Rankl N. B. Gurganus T. M. Marr C. M. Barna J. B. Walters M. M. (1993). A COMPARISON OF LARGE-SCALE SF9 INSECT-CELL GROWTH AND PROTEIN-PRODUCTION - STIRRED VESSEL VS AIRLIFT. Biotechniques 15 (6), 1052–1059. 8292338

[B172] Rim N.-G. Roberts E. G. Ebrahimi D. Dinjaski N. Jacobsen M. M. Martin-Moldes Z. (2017). Predicting silk fiber mechanical properties through multiscale simulation and protein design. Acs Biomaterials Sci. and Eng. 3 (8), 1542–1556. 10.1021/acsbiomaterials.7b00292 PMC5617357 28966980

[B173] Rnjak J. Li Z. Maitz P. K. M. Wise S. G. Weiss A. S. (2009). Primary human dermal fibroblast interactions with open weave three-dimensional scaffolds prepared from synthetic human elastin. Biomaterials 30 (32), 6469–6477. 10.1016/j.biomaterials.2009.08.017 19712968

[B174] Roberts E. G. Rim N.-G. Huang W. Tarakanova A. Yeo J. Buehler M. J. (2018). Fabrication and characterization of recombinant silk-elastin-like-protein (SELP) fiber. Macromol. Biosci. 18 (12), e1800265. 10.1002/mabi.201800265 30417967 PMC6960454

[B175] Rong Y.-H. Zhang G.-A. Wang C. Ning F.-G. (2008). Quantification of type I and III collagen content in normal human skin in different age groups. Zhonghua shao shang za zhi = Zhonghua shaoshang zazhi = Chin. J. burns 24 (1), 51–53. 18512563

[B176] Rosano G. L. Ceccarelli E. A. (2014a). Recombinant protein expression in *Escherichia coli*: advances and challenges. Front. Microbiol. 5, 172. 10.3389/fmicb.2014.00172 24860555 PMC4029002

[B177] Rosano G. L. Ceccarelli E. A. (2014b). Recombinant protein expression in *Escherichia coli*: advances and challenges. Front. Microbiol. 5, 172. 10.3389/fmicb.2014.00172 24860555 PMC4029002

[B178] Rutschmann C. Baumann S. Cabalzar J. Luther K. B. Hennet T. (2014). Recombinant expression of hydroxylated human collagen in *Escherichia coli* . Appl. Microbiol. Biotechnol. 98 (10), 4445–4455. 10.1007/s00253-013-5447-z 24362857

[B179] Sahdev S. Khattar S. K. Saini K. S. (2008). Production of active eukaryotic proteins through bacterial expression systems: a review of the existing biotechnology strategies. Mol. Cell Biochem. 307 (1-2), 249–264. 10.1007/s11010-007-9603-6 17874175

[B180] Salehi S. Koeck K. Scheibel T. (2020). Spider silk for tissue engineering applications. Molecules 25 (3), 737. 10.3390/molecules25030737 32046280 PMC7037138

[B181] Santos F. V. d. Siqueira R. L. Ramos L. d. M. Yoshioka S. A. Branciforti M. C. Correa D. S. (2024). Silk fibroin-derived electrospun materials for biomedical applications: a review. Int. J. Biol. Macromol. 254, 127641. 10.1016/j.ijbiomac.2023.127641 37913875

[B182] Santos M. Serrano-Ducar S. Gonzalez-Valdivieso J. Vallejo R. Girotti A. Cuadrado P. (2019). Genetically engineered elastin-based biomaterials for biomedical applications. Curr. Med. Chem. 26 (40), 7117–7146. 10.2174/0929867325666180508094637 29737250

[B183] Sarangthem V. Singh T. D. Dinda A. K. (2021). Emerging role of elastin-like polypeptides in regenerative medicine. Adv. Wound Care 10 (5), 257–269. 10.1089/wound.2019.1085 PMC8024241 32602815

[B184] Schillberg S. Finnern R. (2021). Plant molecular farming for the production of valuable proteins ? Critical evaluation of achievements and future challenges. J. Plant Physiology 258, 153359. 10.1016/j.jplph.2020.153359 33460995

[B185] Schmelzer C. E. H. Hedtke T. Heinz A. (2020). Unique molecular networks: formation and role of elastin cross-links. Iubmb Life 72 (5), 842–854. 10.1002/iub.2213 31834666

[B186] Schnieke A. Dziadek M. Bateman J. Mascara T. Harbers K. Gelinas R. (1987). Introduction of the human pro alpha 1(I) collagen gene into pro alpha 1(I)-deficient Mov-13 mouse cells leads to formation of functional mouse-human hybrid type I collagen. Proc. Natl. Acad. Sci. U. S. A. 84 (3), 764–768. 10.1073/pnas.84.3.764 3468512 PMC304296

[B187] Shadrina D. V. Venediktov A. A. Evdokimov S. V. Vaskovskii V. A. Artyukhina E. A. Revishvili A. S. (2020). Assessment of biocompatibility and local action of biomaterial for production of an envelope for implanted heart electronic devices. Bull. Exp. Biol. Med. 168 (3), 375–377. 10.1007/s10517-020-04712-0 31938920

[B188] Shanmugaraj B. Bulaon I. J. C. Phoolcharoen W. (2020). Plant molecular farming: a viable platform for recombinant biopharmaceutical production. Plants-Basel 9 (7), 842. 10.3390/plants9070842 32635427 PMC7411908

[B189] Shaoping C. Li S. Yongwen Q. I. N. Zailong C. A. I. (2005). Construction of a eukaryotic expression vector hVEGF165-fused hirudin/pcDNA3.0 and its expression. Acad. J. Second Mil. Med. Univ. 26 (9), 983–987.

[B190] Sharma S. Kaur R. Singh A. (2017). Recent advances in CRISPR/Cas mediated genome editing for crop improvement. Plant Biotechnol. Rep. 11 (4), 193–207. 10.1007/s11816-017-0446-7

[B191] Shayegan M. Altindal T. Kiefl E. Forde N. R. (2016). Intact telopeptides enhance interactions between collagens. Biophysical J. 111 (11), 2404–2416. 10.1016/j.bpj.2016.10.039 PMC5153562 27926842

[B192] Shi J. Gao Y. He J. Ma X. (2017b). Recombinant coexpression for hydroxylated human type Ⅲ collagen alpha chain with a viral prolyl 4-hydroxylase in Pichia pastoris. J. Northwest Univ. Nat. Sci. Ed. 47 (2), 231–236.

[B193] Shi J. Ma X. Gao Y. Fan D. Zhu C. Mi Y. (2017a). Hydroxylation of human type III collagen alpha chain by recombinant coexpression with a viral prolyl 4-hydroxylase in *Escherichia coli* . Protein J. 36 (4), 322–331. 10.1007/s10930-017-9723-0 28589291

[B194] Shi P. Lin Y. A. Pastuszka M. Cui H. Mackay J. A. (2014). Triggered sorting and co-assembly of genetically engineered protein microdomains in the cytoplasm. Adv. Mater 26 (3), 449–454. 10.1002/adma.201303356 24136711 PMC3947098

[B195] Shin W. Kim J. S. Kim H. Choi H. J. Lee H. J. Um M. K. (2021). Material design for 3D multifunctional hydrogel structure preparation. Macromol. Mater. Eng. 306 (5). 10.1002/mame.202100007

[B196] Shoulders M. D. Raines R. T. (2009). Collagen structure and stability. Annu. Rev. Biochem. 78, 929–958. 10.1146/annurev.biochem.77.032207.120833 19344236 PMC2846778

[B197] Specks U. Mayer U. Nischt R. Spissinger T. Mann K. Timpl R. (1992). STRUCTURE OF RECOMBINANT N-TERMINAL GLOBULE OF TYPE-VI COLLAGEN ALPHA-3 CHAIN AND ITS BINDING TO HEPARIN AND HYALURONAN. Embo J. 11 (12), 4281–4290. 10.1002/j.1460-2075.1992.tb05527.x 1425570 PMC557001

[B198] Steplewski A. Hintze V. Fertala A. (2007). Molecular basis of organization of collagen fibrils. J. Struct. Biol. 157 (2), 297–307. 10.1016/j.jsb.2006.10.009 17126032

[B199] Suzuki Y. (2016). Structures of silk fibroin before and after spinning and biomedical applications. Polym. J. 48 (11), 1039–1044. 10.1038/pj.2016.77

[B200] Tanaka C. Asakura T. (2009). Synthesis and characterization of cell-adhesive silk-like proteins constructed from the sequences of anaphe silk fibroin and fibronectin. Biomacromolecules 10 (4), 923–928. 10.1021/bm801439t 19236090

[B201] Toman P. D. Pieper F. Sakai N. Karatzas C. Platenburg E. de Wit I. (1999). Production of recombinant human type I procollagen homotrimer in the mammary gland of transgenic mice. Transgenic Res. 8 (6), 415–427. 10.1023/a:1008959924856 10767986

[B202] Tripathi N. K. Shrivastava A. (2019). Recent developments in bioprocessing of recombinant proteins: expression hosts and process development. Front. Bioeng. Biotechnol. 7, 420. 10.3389/fbioe.2019.00420 31921823 PMC6932962

[B203] Tzaphlidou M. Zervakis M. (2004). Aged skin: detection of alterations of major collagen types ratio by image processing of electron-optical data. Micron 35 (3), 221–225. 10.1016/j.micron.2003.11.008 15036276

[B204] Urry D. W. Hugel T. Seitz M. Gaub H. E. Sheiba L. Dea J. (2002). Elastin: a representative ideal protein elastomer. Philos. Trans. R. Soc. Lond B Biol. Sci. 357 (1418), 169–184. 10.1098/rstb.2001.1023 11911774 PMC1692938

[B205] Urry D. W. Parker T. M. Reid M. C. Gowda D. C. (1991). Biocompatibility of the bioelastic materials, poly(GVGVP) and its γ-irradiation cross-linked matrix: summary of generic biological test results. J. Bioact. Compatible Polym. 6 (3), 263–282. 10.1177/088391159100600306

[B206] van Eldijk M. B. Wang J. C. Y. Minten I. J. Li C. Zlotnick A. Nolte R. J. M. (2012). Designing two self-assembly mechanisms into one viral capsid protein. J. Am. Chem. Soc. 134 (45), 18506–18509. 10.1021/ja308132z 23101937 PMC3510441

[B207] Vaughan P. R. Galanis M. Richards K. M. Tebb T. A. Ramshaw J. A. M. Werkmeister J. A. (1998). Production of recombinant hydroxylated human type III collagen fragment in *Saccharomyces cerevisiae* . DNA Cell Biol. 17 (6), 511–518. 10.1089/dna.1998.17.511 9655244

[B208] Vendrely C. Scheibel T. (2007). Biotechnological production of spider-silk proteins enables new applications. Macromol. Biosci. 7 (4), 401–409. 10.1002/mabi.200600255 17429812

[B209] Vindin H. Mithieux S. M. Weiss A. S. (2019). Elastin architecture. Matrix Biol. 84, 4–16. 10.1016/j.matbio.2019.07.005 31301399

[B210] Voller L. Rahman Z. (2023). Translational biochemistry of the skin. Facial Plastic Surg. Clin. N. Am. 31 (4), 443–452. 10.1016/j.fsc.2023.06.009 37806678

[B211] Vuorela A. Myllyharju J. Pihlajaniemi T. Kivirikko K. I. (1999). Coexpression with collagen markedly increases the half-life of the recombinant human prolyl 4-hydroxylase tetramer in the yeast Pichia pastoris. Matrix Biol. 18 (5), 519–522. 10.1016/s0945-053x(99)00040-2 10601739

[B212] Wang H. Shi Y. Wang L. Yang Z. (2013). Recombinant proteins as cross-linkers for hydrogelations. Chem. Soc. Rev. 42 (3), 891–901. 10.1039/c2cs35358j 23169442

[B213] Wang J. Qiu H. Xu Y. Gao Y. Tan P. Zhao R. (2022a). The biological effect of recombinant humanized collagen on damaged skin induced by UV-photoaging: an *in vivo* study. Bioact. Mater. 11, 154–165. 10.1016/j.bioactmat.2021.10.004 34938920 PMC8665261

[B214] Wang J.-N. Yan S.-Q. Lu C.-D. Bai L. (2009). Biosynthesis and characterization of typical fibroin crystalline polypeptides of silkworm *Bombyx mori* . Mater. Sci. and Eng. C-Biomimetic Supramol. Syst. 29 (4), 1321–1325. 10.1016/j.msec.2008.10.029

[B215] Wang K. Ji L. Hua Z. (2017). Functional peptides from laminin-1 improve the cell adhesion capacity of recombinant mussel adhesive protein. Protein Peptide Lett. 24 (4), 348–352. 10.2174/0929866524666170123142225 28117011

[B216] Wang S. Zhang Y. Yang M. Ye L. Gong L. Qian Q. (2016). Characterization of transgenic silkworm yielded biomaterials with calcium-binding activity. Plos One 11 (7), e0159111. 10.1371/journal.pone.0159111 27414647 PMC4944971

[B217] Wang S. Zhu H. Meng Q. (2022b). Preparation and characterization of nanofibrous membranes electro-spun from blended poly(l-lactide-co-ε-caprolactone) and recombinant spider silk protein as potential skin regeneration scaffold. Int. J. Mol. Sci. 23 (22), 14055. 10.3390/ijms232214055 36430534 PMC9698895

[B218] Wang X. Liang Q. Luo Y. Ye J. Yu Y. Chen F. (2024). Engineering the next generation of theranostic biomaterials with synthetic biology. Bioact. Mater. 32, 514–529. 10.1016/j.bioactmat.2023.10.018 38026437 PMC10660023

[B219] Weitzhandler I. Dzuricky M. Hoffmann I. Quiroz F. G. Gradzielski M. Chilkoti A. (2017). Micellar self-assembly of recombinant resilin-/elastin-like block copolypeptides. Biomacromolecules 18 (8), 2419–2426. 10.1021/acs.biomac.7b00589 28570078 PMC6364677

[B220] Wise S. G. Mithieux S. M. Weiss A. S. (2009). Silk biomaterials in wound healing and skin regeneration therapeutics: from bench to bedside. Acta Biomater. 103, 24–51. 10.1016/j.actbio.2019.11.050 31805409

[B221] Wongpinyochit T. Johnston B. F. Seib F. P. (2018). Degradation behavior of silk nanoparticles-enzyme responsiveness. Acs Biomaterials Sci. and Eng. 4 (3), 942–951. 10.1021/acsbiomaterials.7b01021 33418776

[B222] Work R. W. (1985). Viscoelastic behaviour and wet supercontraction of major ampullate silk fibres of certain orb-web-building spiders (Araneae). J. Exp. Biol. 118, 379–404. 10.1242/jeb.118.1.379

[B223] Wu Y. Kang Z. Tian Z. Wu M. Wang J. (2017). Biosynthesis and characterization of recombinant silk-like polypeptides derived from the heavy chain of silk fibrion. Polymers 9 (12), 669. 10.3390/polym9120669 30965969 PMC6418719

[B224] Xia X.-X. Qian Z.-G. Ki C. S. Park Y. H. Kaplan D. L. Lee S. Y. (2010). Native-sized recombinant spider silk protein produced in metabolically engineered *Escherichia coli* results in a strong fiber. Proc. Natl. Acad. Sci. U. S. A. 107 (32), 14059–14063. 10.1073/pnas.1003366107 20660779 PMC2922564

[B225] Xiao Y. Ling S. Pei Y. (2021). Structure of elastin. Methods Mol. Biol. 2347, 27–33. 10.1007/978-1-0716-1574-4_3 34472052

[B226] Xie H. Lucchesi L. Zheng B. Ladich E. Pineda T. Merten R. (2017). Treatment of burn and surgical wounds with recombinant human tropoelastin produces new elastin fibers in scars. J. Burn Care and Res. 38 (5), E859–E867. 10.1097/bcr.0000000000000507 28221299

[B227] Xin L. Renhuai Z. Zili L. Huawei A. Dong L. Bo L. (2020). Isolation and purification of recombinant human type III collagen. Food Ferment. Industries (16), 159–163.

[B228] Xiong L. Zhou C. Tong L. Han X. Zou Y. Dong Z. (2023). Injectable hydrogels of recombinant human collagen type III and chitosan with antibacterial and antioxidative activities for wound healing. J. Mater. Chem. B 11 (18), 4131–4142. 10.1039/d3tb00176h 37114495

[B229] Xu H. (2014). The advances and perspectives of recombinant protein production in the silk gland of silkworm *Bombyx mori* . Transgenic Res. 23 (5), 697–706. 10.1007/s11248-014-9826-8 25113390

[B230] Xu J. Wang L. N. Zhu C. H. Fan D. D. Ma X. X. Mi Y. (2015). Co-expression of recombinant human prolyl with human collagen *α*1 (III) chains in two yeast systems. Lett. Appl. Microbiol. 61 (3), 259–266. 10.1111/lam.12447 26031396

[B231] Xu T. Zhang J. Wang T. Wang X. (2022). Recombinant antibodies aggregation and overcoming strategies in CHO cells. Appl. Microbiol. Biotechnol. 106 (11), 3913–3922. 10.1007/s00253-022-11977-0 35608667

[B232] Yamamoto E. Kawamura Y. (2020). Biomimetic materials for the regeneration of extracellular matrix in biological soft tissues. J. Photopolym. Sci. Technol. 33 (2), 235–240. 10.2494/photopolymer.33.235

[B233] Yan Z. Liu S. Liu A.-Q. Wang H.-Y. (2022). The oral administration of elastin peptide reduces ultraviolet light-induced photoaging in hairless mice. Pak. J. Zoology 54 (1), 153–160. 10.17582/journal.pjz/20200629020649

[B234] Yang C. L. Hillas P. J. Báez J. A. Nokelainen M. Balan J. Tang J. (2004). The application of recombinant human collagen in tissue engineering. Biodrugs 18 (2), 103–119. 10.2165/00063030-200418020-00004 15046526

[B235] Yang G. Wu M. Yi H. Wang J. (2016). Biosynthesis and characterization of a non-repetitive polypeptide derived from silk fibroin heavy chain. Mater. Sci. and Eng. C-Materials Biol. Appl. 59, 278–285. 10.1016/j.msec.2015.10.023 26652374

[B236] Yang Y. Xu R. Wang C. Guo Y. Sun W. Ouyang L. (2022). Recombinant human collagen-based bioinks for the 3D bioprinting of full-thickness human skin equivalent. Int. J. Bioprinting 8 (4), 611–160. 10.18063/ijb.v8i4.611 PMC9668586 36404779

[B237] Yang Y. J. Kim C. S. Choi B.-H. Cha H. J. (2015). Mechanically durable and biologically favorable protein hydrogel based on elastic silklike protein derived from sea anemone. Biomacromolecules 16 (12), 3819–3826. 10.1021/acs.biomac.5b01130 26539814

[B238] Yang Y. X. Li Q. Li W. D. Wang T. Y. Feng H. G. (2023). Factors and mechanisms affecting the secretion of recombinant protein in CHO cells. Curr. Pharm. Biotechnol. 24 (3), 391–400. 10.2174/1389201023666220603121316 35658884

[B239] Ye J. Xie C. Wang C. Huang J. Yin Z. Heng B. C. (2021). Promoting musculoskeletal system soft tissue regeneration by biomaterial-mediated modulation of macrophage polarization. Bioact. Mater. 6 (11), 4096–4109. 10.1016/j.bioactmat.2021.04.017 33997496 PMC8091177

[B240] Yigit S. Dinjaski N. Kaplan D. L. (2016). Fibrous proteins: at the crossroads of genetic engineering and biotechnological applications. Biotechnol. Bioeng. 113 (5), 913–929. 10.1002/bit.25820 26332660

[B241] Yin Z. Hu J.-j. Yang L. Zheng Z.-f. An C.-r. Wu B.-b. (2016). Single-cell analysis reveals a nestin(+) tendon stem/progenitor cell population with strong tenogenic potentiality. Sci. Adv. 2 (11), e1600874. 10.1126/sciadv.1600874 28138519 PMC5262457

[B242] Yousefpour P. Ni K. Irvine D. J. (2023). Targeted modulation of immune cells and tissues using engineered biomaterials. Nat. Rev. Bioeng. 1 (2), 107–124. 10.1038/s44222-022-00016-2 37772035 PMC10538251

[B243] Yu-han Z. Jing-xuan Z. Nan-hai Z. Liang Z. Lie-bing Z. Feng Z. (2023). Advances in ameliorating effects of collagen peptides and elastin peptides on photoaged skin. Food Res. Dev. 44 (11), 208–216. 10.12161/j.issn.1005-6521.2023.11.030

[B244] Zhang J. Tang Q. Zhou A. Yang S. (2015). Research progress of collagen-based three-dimensional porous scaffolds used in skin tissue engineering. J. Biomed. Eng. 32 (4), 924–928. 26710471

[B245] Zhang W. Fan Y. (2021). Structure of animal silks. Methods Mol. Biol. Clift. N.J. 2347, 3–15. 10.1007/978-1-0716-1574-4_1 34472050

[B246] Zhao B. Li N. K. Yingling Y. G. Hall C. K. (2016). LCST Behavior is Manifested in a Single Molecule: elastin-Like polypeptide (VPGVG)_<i>n</i>_ . Biomacromolecules 17 (1), 111–118. 10.1021/acs.biomac.5b01235 26595324

[B247] Zhou C. Z. Confalonieri F. Jacquet M. Perasso R. Li Z. G. Janin J. (2001). Silk fibroin: structural implications of a remarkable amino acid sequence. Proteins-Structure Funct. Genet. 44 (2), 119–122. 10.1002/prot.1078 11391774

[B248] Zhou J. He W. Luo G. Wu J. (2013). Fundamental immunology of skin transplantation and key strategies for tolerance induction. Archivum Immunol. Ther. Exp. 61 (5), 397–405. 10.1007/s00005-013-0233-2 23685832

[B249] Zhou Y. Raju R. Alves C. Gilbert A. (2018). Debottlenecking protein secretion and reducing protein aggregation in the cellular host. Curr. Opin. Biotechnol. 53, 151–157. 10.1016/j.copbio.2018.01.007 29414073

[B250] Zhu H. N. Rising A. Johansson J. Zhang X. H. Lin Y. Zhang L. (2020). Tensile properties of synthetic pyriform spider silk fibers depend on the number of repetitive units as well as the presence of N- and C-terminal domains. Int. J. Biol. Macromol. 154, 765–772. 10.1016/j.ijbiomac.2020.03.042 32169447

[B251] Zorina A. Zorin V. Kudlay D. Kopnin P. (2022). Molecular mechanisms of changes in homeostasis of the dermal extracellular matrix: both involutional and mediated by ultraviolet radiation. Int. J. Mol. Sci. 23 (12), 6655. 10.3390/ijms23126655 35743097 PMC9223561

